# HIV‐Exposed Seronegative Female Sex Workers Show Different Cellular Immune Profiles Across the Menstrual Cycle

**DOI:** 10.1111/aji.70198

**Published:** 2025-12-19

**Authors:** Monika M. Kowatsch, Kenneth Omollo, Frideborg Bradley, Anna Månberg, Peter Nilsson, Sofia Bergström, Julius Oyugi, Joshua Kimani, Kristina Broliden, Keith R. Fowke, Julie Lajoie

**Affiliations:** ^1^ Department of Medical Microbiology and Infectious Diseases University of Manitoba Winnipeg Canada; ^2^ Department of Medical Microbiology and Immunology University of Nairobi Nairobi Kenya; ^3^ Department of Medicine Solna, Division of Infectious Diseases, Karolinska Institutet, Department of Infectious Diseases Karolinska University Hospital, Center for Molecular Medicine Stockholm Sweden; ^4^ Kungliga Tekniska Hogskolan Institutionen For Proteinvetenskap Stockholm Stockholm Sweden; ^5^ Partner For Health and Development in Africa University of Nairobi Nairobi Kenya

## Abstract

**Problem:**

Female sex workers (FSWs) are at higher risk of acquiring HIV. Interestingly, some FSWs who are highly exposed remain seronegative for HIV (HESN). This natural resistance to HIV infection has been attributed to an immune quiescence (IQ) phenotype. Our study investigates how the menstrual cycle phases (follicular and luteal) impact the immune responses in Kenyan FSWs.

**Methods:**

This is a part of the Longitudinal Assessment of Mucosal Immune Quiescence study (LAMIQ), 48 FSWs not living with HIV and not using hormonal contraception were followed for a menstrual cycle and divided into two groups based on duration of sex work: New Negative (NN) with 3 years or less and HESN with at least 7 years of involvement in sex work. We obtained blood and cervicovaginal samples and measured sex hormone, cytokine, and chemokine levels, and blood and endocervical T‐cell and NK‐cell phenotypes.

**Results:**

We observed differences in how the immune response of NN and HESN responds to sex hormones. Indeed, the level of mucosal Annexin A3 measured was higher during the luteal phase in HESN, which was not observed in NN. HESN exhibited a higher CD39 expression on their Treg during the luteal phase, while maintaining CTLA‐4 expression compared to NN. Furthermore, in HESN, NK cell activation varied across the menstrual cycle phases. They had a higher expression of NKG2D and an increase in the cluster of CD95+ HLA‐DR+ NK cells during the follicular phase. This suggests stronger innate immune activation in HESN during the follicular phase of the menstrual cycle.

**Conclusion:**

Our data indicate that, in HESN, there is a modulation of the immune response based on the menstrual cycle, which potentially limits the availability of  HIV target cells at the female genital tract during the luteal phase of the menstrual cycle (window of susceptibility).

## 1 Introduction

Many social and biological factors contribute to why women are at greater risk of acquiring HIV than men during vaginal sex. [1]. In fact, in 2023, women and young adolescents in Africa made up 62% of all new HIV infections [1]. Despite this increased susceptibility, there has been relatively little research into how the menstrual cycle might influence the immune system—and how that, in turn, could affect susceptibility to HIV.

The female genital tract immune response must be able to respond to an array of pathogens and, at the same time, must allow for reproductive function (supporting sperm migration, fertilization, and implantation). These functions are especially controlled by sex hormones. The expression of sex hormones (progesterone and estrogen) fluctuates across the menstrual cycle, and while progesterone is mainly expressed during the luteal phase, estrogen expression fluctuates throughout the menstrual cycle and reaches its highest levels during the follicular phase of the menstrual cycle. Together, they regulate the fluctuations of immune proteins, epithelial barrier thickness, and the viscosity of the vaginal mucus [2, 3]. High levels of estrogen have been linked to looser tight junctions [4], a factor that, at least theoretically, could make it easier for HIV to establish an infection after an exposure. Progesterone expression increases during the luteal phase of the menstrual cycle and is associated with an anti‐inflammatory Th2 cytokine response [5]. In sexually active women, a higher level of progesterone has been linked with increased TNF‐α expression, an effect not seen in women who are not sexually active [5]. This points to a potential interaction between sexual activity and how the immune system responds to hormones. Generally, in vitro and animal studies have associated estrogen with a protective effect against HIV‐1. In contrast, progesterone and progestin‐based contraception, like Depot Medroxyprogesterone acetate, have been associated with an increased risk of HIV susceptibility [6, 7, 8]. Because progesterone possesses anti‐inflammatory properties, and thins the vaginal epithelial and alters the cervical mucus, it has been hypothesized that the luteal phase of the menstrual cycle (in which the level of progesterone is high) represents the window of susceptibility for HIV infection [9]

However, despite the impact that sex hormones can have on the immune response, we know surprisingly little about how the menstrual cycle affects the immune systems of female sex workers (FSWs), who face disproportionately high HIV risk. Our study aims to help close this gap by examining the immune responses during the follicular and luteal phases in FSWs.

While FSWs are generally more exposed to HIV, not all are equally susceptible. Our earlier work has shown that the mucosal immune responses evolve with time in sex work, trending toward a less inflammatory state with years of sex work [10]. This shift begins within the first year and becomes more pronounced with longer involvement in sex work [10].

To establish an infection, HIV preferentially infects activated CD4+ T cells [11]. While quiescent cells can be infected by the virus, in vitro studies show that it leads to inefficient viral replication [12, 13]. In Kenya, one group of FSWs from the Puwmani cohort has been identified as being naturally resistant to HIV infection. This group of highly exposed seronegative (HESN) women represents an extreme phenotype of HESN, as they have been involved in sex work for over 7 years. In this cohort, the HESN phenotype has been associated with a distinct immune profile named Immune Quiescence (IQ). The IQ phenotype has been associated with a lower baseline level of blood and mucosal activated T cells, a higher proportion of blood regulatory T cells, lower mucosal expression of pro‐inflammatory chemokines MIG and IP‐10, and a higher level of anti‐proteases in the female genital tract, which altogether creates a mucosal environment less favorable for HIV infection [14, 15, 16].

However, even though sex hormones play an important role in the immune response, we still don't know if the immune system of HIV‐exposed seronegative (HESN) women and those newer to sex work are affected the same way by sex hormones. To answer this question, we conducted a longitudinal study to characterize the blood and genital NK and T cell populations of FSWs who are not using hormonal contraceptives during the follicular and luteal phases of the menstrual cycle. Our findings suggest that HESN women modulate the immune response differently according to the phase of the menstrual cycle, which could reduce the availability of HIV target cells during the luteal phase.

## 2 Method Section

Our study is part of the Longitudinal Assessment of Mucosal Immune Quiescence study (LAMIQ) [17, 18]. The LAMIQ study followed participants for three months (bi‐monthly sampling). During the first month, participants came to the clinics twice: once to target the follicular phase (3–5 days post menses) and 14 to 20 days later to target the luteal phase of the menstrual cycle. During the first month, participants were active in sex work. During the second month, participants were asked to take a break from sex work, and in the third month, they resumed sexual activities and sex work. The data, presented herein, are from the first two visits of the study, representing one menstrual cycle. The participants were divided into two groups based on their duration of sex work, as outlined in Table 1. We included in this analysis 25 FSWs newly involved in sex work (≤ 3 years) [new negative (NN)] and 23 women with > 7 years in sex work [HIV exposed seronegative (HESN)]. Informed consent was obtained from all participants. The LAMIQ study was approved by the ethical review boards from the University of Manitoba, the regional Ethical Review Board in Stockholm, and the Kenyatta National Hospital/University of Nairobi.

**TABLE 1:** Sociodemographics for all groups included in the study by menstrual phase.

	HESN		New Neg	
Parameter	Follicular phase	Luteal phase	Follicular phase	Luteal phase
Number at each visit	23	22	25	19
Age (years)	36^a^ [33.5–38.5]	36^a^ [33–40.5]	32^a^ [24–34]	31^a^ [24.5–37.5]
Number of clients in last 7 days	5 [3–9]	5 [3.3–10]	5 [2–7]	4 [2.5–5.8]
BV				
Normal	10 (62.5)	9 (40.9)	11 (44.0)	11 (61.1)
Intermediate	6 (27.2)	7 (31.8)	6 (24.0)	4 (22.2)
Positive	6 (27.2)	6 (27.3)	8 (32.0)	3 (16.6)
Duration of sex work (months)	10^a^ [8.5–11.5]	10^a^ [9–11.5]	2^a^ [1.5–3]	2^a^ [1.17–3]
Normal cycle length				
26 to 32 days	13 (56.5)	11 (50.0)	13 (52.0)	13 (68.4)
< 26 or > 32 days	10 (43.5)	11 (50.0)	12 (48.0)	6 (31.6)
Ratio blood CD4: CD8	3.06 [2.1–3.7]	2.75 [2.1–5.7]	2.23 [2–3.25]	3 [1.2–6.2]
Ratio cervical CD4: CD8	1.52 [1.4–2.5]	2.49 [1.8–3.8]	1.71 [0.9–2.8]	1.76 [1.1–1.8]
Estradiol (pg/mL)	130.0 [60.0–180.0]	125.0 [55.0–262.5]	180.0 [100.0–270.0]	280.0 [112.5–367.5]
Progesterone (pg/mL)	1050^b^ [715–1514]	5645^b^ [1962–7960]	1140^b^ [620–2142]	6280^b^ [2440–11345]

*Note:* Data is presented at median [IQR] or N (%).

^a^ *p* value < 0.05 between HESN and New Neg women at both follicular and luteal phases.

^b^ *p* values < 0.001 between follicular and luteal phases.

The exclusion and inclusion criteria have been described previously [17, 19]. Briefly, participants could be enrolled if they were between 18 and 40 years of age, were pre‐menopausal (based on self‐declaration and having regular menstrual cycles during the study) with no prior hysterectomy, were not pregnant or breastfeeding, and had a negative test for *Neisseria gonorrhoeae*, *Chlamydia trachomatis*, and syphilis infection. HSV‐2 status was not determined. There was no sign of active infections at the time of sampling. All participants answered a demographic and behavioral questionnaire. An HIV rapid test was done at the beginning and at the end of the study (Determine, Inverness Medical, Japan). Bacterial vaginosis (BV) was defined using the Nugent score. *Trichomonas vaginalis* (TV) was diagnosed using standard saline microscopy. Urine samples were collected for PCR detection of *Neisseria gonorrhea* (NG) and *Chlamydia trachomatis* (CT) (Roche Amplicor kits). Furthermore, for this sub‐study, only women whose visit 1 was during the follicular phase of the menstrual cycle and their visit 2 during the luteal stage of their menstrual cycle were analyzed.

Following enrolment, women were asked to return to the clinic 3–5 days after their last menses, to match the follicular phase of the menstrual cycle. Then, they were asked to come back to the clinics two weeks later for visit 2, which targeted the luteal phase of the menstrual cycle. Participants were followed in the same way over the next two months. We confirmed the phase of the menstrual cycle with different strategies. At each visit, women reported the dates of their last menses. The length of their cycle was calculated based on the average of three consecutive cycles. The start of the luteal phase was defined as 14 days before the end of the menstrual cycle. While the main study aimed to target visit 1 during the follicular phase and visit 2 during the luteal phase of the menstrual cycle, some women did not fit this pattern. Only women in the follicular phase at the first visit and in the luteal phase at the second visit were included herein, resulting in 57 visits during the follicular phase and 41 during the luteal phase. Estrogen and progesterone levels were calculated at both phases by a multiplex bead array.

### 2.1 Sample Collection and Processing

Blood (Peripheral blood mononuclear cells (PBMCs) and plasma) and Cervicovaginal samples (cervicovaginal lavage (CVL) and cervical mononuclear cells (CMCs)) were collected as described previously [20, 21]. Fresh cervical cells and PBMC (106 cells/donor) were stained for *ex vivo* phenotyping using three separate flow panels (Supplemental Table S1 and Supplemental Figure S1). The first one was designed to characterize T cell activation in PBMC and CMC. Phenotyping: PE.Cy5‐CD3 (clone UCHT1) (BD Biosciences, USA), V500‐CD8 (BD Biosciences, USA), FITC‐CD4 (clone RPA‐T4) (BD Biosciences, USA), APC‐CD161 (clone DX12) (BD Biosciences, USA); HIV co‐receptor: V450‐CCR5 (clone 2D7/CCR5) (BD Biosciences, USA); activation markers: PE.Cy7‐CD69  (clone FN50)(BD Biosciences, USA), PE‐CD95 (DX2) (BD Biosciences, USA), APC.H7‐HLA‐DR (clone L243) (BD Biosciences, USA); live cells Far Red‐Live Dead discriminant (Invitrogen, USA). Treg cells were defined in PBMC. Markers used to define Treg populations: V500‐CD3 (UCHT1) (BD), APC‐Cy7‐CD4 (SK3) (BioLegend), PE‐CF594‐CD25 (M0A251)BD), PeCy7‐CD127(HIL‐7R‐M21)(BD), PE‐FoxP3(PCH101)(eBioscience); activation: APC‐Helios (22F6) (eBioscience), PECy5‐CTLA‐4(BNI3)(BD), BV421‐CD39 (A1) (BioLegend), FITC‐Integrin β7(F1B504) (BD); live cells: Far Red Live Dead discriminant. NK cells were characterized from PBMC samples: Pe‐Cy5‐CD45 (HI30) (BD), V500‐CD3(UCHT1)(BD), CD14 (M5E2) (BD), CD19 (HIB19) (BD)(dump), Alexa‐700‐CD16 (3G8) (BD), Pe‐Cy‐CD56 (B159) (BD), APC‐CD62L (DREG‐56) (BioLegend), FITC‐CD57 (NK‐1) (BD), PE‐CD95 (DX2) (BD), APC‐h7‐HLA‐DR (L243) (BD), PE‐CF594‐NKG2D (NKL) (BD), V450‐CCR5 (2D7/CCR5) (BD), and Far red‐Live Dead discriminant. Samples were acquired on an LSRII flow cytometer (BD System, USA) and analyzed using FlowJo v10.0.8r1 (TreeStar, USA). As we described previously, cervico mononuclear samples with less than 100 live CD3+ T cells were excluded from the analysis [21].

The concentrations of pro‐inflammatory cytokines and chemokines in the cervicovaginal and plasma were measured by Milliplex (Millipore, Merck KGaA, Darmstadt, Germany) according to the manufacturer's instructions and analyzed on the BioPlex‐200 (Bio‐Rad, Mississauga, ON, Canada). All samples were run in duplicate, and the mean of both samples was used for the analyses. CVL samples were incubated following the overnight protocol as previously described [14]. For samples below the limit of detection, an assigned value of half the manufacturer's specified detection limit was used.

Progesterone and estrogen levels were measured in plasma‐extracted hormones using the MILLIPLEX MAP Steroid/Thyroid Hormone Magnetic Bead Panel (Millipore, Merck KGaA, Darmstadt, Germany) following the manufacturer's protocol.

Protein profiling on CVL samples was performed using a suspension bead array of human protein atlas (HPA) antibodies as previously described [22]. Target proteins were selected from a larger panel of proteins involved in inflammation and HIV resistance in the female genital tract [22] and complemented with additional proteins associated with inflammation and sex hormones. This resulted in 61 antibodies directed towards 50 proteins (Supplemental Table S2). Briefly, CVL samples were randomized, diluted, biotinylated, and heat‐treated together with twelve technical replicates. The bead array was distributed in a 384‐well microtiter plate. Samples were transferred and incubated overnight at ambient temperature. Subsequent washing, crosslinking, and detection were performed. For each sample, at least 50 beads of each identity were measured in a FlexMap 3D instrument (Luminex Corp., Austin, United States). The results were displayed as median fluorescence intensity (MFI) in arbitrary units.

### 2.2 Statistical Methods

All the statistical analysis and the figures were generated using RStudio (version 2023.09.1+494). We tested for Gaussian distribution using the Shapiro–Wilk test, and for the variance between visits we used the Fligner–Killeen Test of Homogeneity of Variances. Raw data were then transformed using either a log transformation for cytokine and expression data or a logit transformation for proportion data. Mixed‐effects models were used to assess the differences between groups and visits (follicular vs. luteal phases) and to account for repeated measures among participants. All analyses were controlled for age. For analyses between HESN and NN, we also controlled for the length of the menstrual cycle as a binary variable: regular (between 26 and 32 days) or abnormal ( < 26 or > 32 days). The false discovery rate was applied using the Benjamini and Hochberg correction by variable group (Supplemental Table S2). As our study was discovery‐based, had a small sample size, and there are limited data on the menstrual cycle's impact on FSWS, we kept the results with an adjusted *p* value < 0.075.

Most of the flow analysis data is based on conventional gating. Multidimensional reduction was also employed to further phenotype the NK subset to allow for characterization of potential transitional NK cell subsets beyond CD16++CD56+ and CD16+CD56++ using a generalized approach to reduce the dimensionality in flow cytometry. It is a multi‐step process, which includes 1) cleaning of raw data to isolate living NK cells as outlined in Supplemental Figures S1 and S2. Applying standardized reduction procedures [23, 24, 25, 26] on the same number of cells from each sample. Briefly, FCS files were downsampled (same number of events/sample) and concatenated at 637 per participant. Following this, three reduction algorithms were applied: tSNE, UMAP, and TriMAP [27] at 1000 iterations/sample.  As the aim of this analysis was to characterize a potential transitional phenotype, the X‐Shift clustering algorithm was used to determine the cell identities present within the study, as it produced the most robust results for our given sample size and event number [23, 24, 25, 26]. Cluster identities were assigned using MFI of each marker per cluster (Supplemental Figure S2a, b) and marker enrichment modeling, which scores each marker by its relative enrichment within the sample [28]. The reduction and clustering outcomes can be found in Supplemental Figure S2. The resulting proportion of each cluster present in each sample was exported from FlowJo and analyzed as described above.

## 3 Results

### 3.1 Sociodemographics

Here, we included two groups of FSWs who are not living with HIV and not using any hormonal contraceptives: HESN and  New Neg. Table 1 provides the principal descriptives of the two groups. Participants were followed for one month: Visit 1 (at the follicular phase) and Visit 2 (at the luteal phase). The two groups were similar in the number of clients in the past seven days and in the proportion of women experiencing normal versus abnormal menstrual cycle lengths. Age and duration of sex work were significantly different between HESN and New Neg women (HESN vs. NN: age—follicular *p* = 0.002, luteal *p* = 0.020; duration of sex work—follicular *p* < 0.0001, luteal *p* < 0.0001). However, this was expected based on our group definitions of HESN > 7 years in sex work and NN ≤ 3 years in sex work.  The ≤ 3 years in sex work group was selected because it is during that period that a sex worker is the most at risk of acquiring HIV, and is the most susceptible. It offers a good comparison group of women involved in sex work for many years, but who remained HIV uninfected [29]. All our analyses were controlled for age.

This study aimed to assess 1) the impact of the menstrual cycle on HESN and New Neg women separately and 2) compare the immune response in HESN versus NN according to the menstrual cycle. Mixed‐effects models, controlling for age and menstrual cycle length, were used to perform these analyses.

### 3.2 Soluble Markers Results

Not surprisingly, the level of progesterone measured in the blood was lower during the follicular phase compared to the levels in the luteal phase of the menstrual cycle. This was observed in both groups (*p* < 0.001; padj = 0.002 for both). The level of progesterone at each phase was similar between HESNs and NN (Supplemental Table S2).

We analyzed the expression of 19 cytokines or chemokines in the blood and the CVL. While the expression of some cytokines in the plasma and the CVL showed differences (see Supplemental Table S2), after false discovery corrections, there were no statistical differences in the CVL or plasma between menstrual cycle phases within each group or between the groups.

**FIGURE 1:**
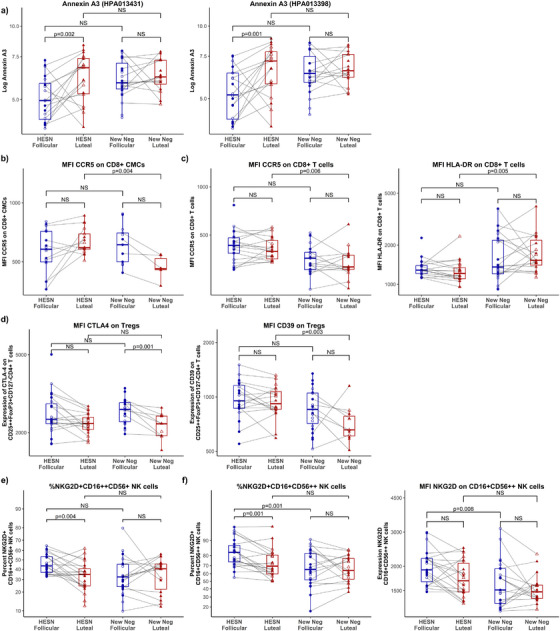
Immune parameters in HESN and New Negs between the follicular and luteal phases of the menstrual cycle. a) **CVL** protein levels of annexin A3 by two separate antibodies (indicated in brackets), b) **CMC**: CD8 CMCs, c) **PBMC**: CD8 T cells in PBMCs, d) **PBMC**: Tregs in PBMCs, e) **PBMC**: CD16++CD56+ NK cells in PBMCs, f) **PBMC**: CD16+CD56++ NK cells in PBMCs. All analyses were performed on the log of parameters due to non‐normal distribution, and mixed‐effects models were used to control for age and cycle length. Solid symbols represent regular cycle lengths (between 26 and 32 days), and open symbols represent abnormal cycle lengths (not between 26 and 32 days). Samples from the follicular phase are in blue circles, and samples from the luteal phase are in red triangles. Un‐adjusted *p* values < 0.05 were considered significant, false discovery rate was applied by sample type, and values < 0.075 were retained and presented here with unadjusted *p* values. A complete list of *p* values and sample types can be found in Supplemental Table S2.

We also performed a mucosal protein profiling analysis of 50 proteins. Of those, only the expression of annexin A3 was altered. Mucosal Annexin A3 levels were higher during the luteal phase compared to the follicular phase in HESN (*p* = 0.001 and *p* = 0.002, respectively; padj = 0.061 for both annexin A3 detecting antibodies (Figure 1a). This difference was not observed in NN across both menstrual cycle phases, and there was no difference in protein expression between HESN and NN women.

### 3.3 T‐Cell Phenotyping

To determine if the immune activation profile was affected by the phases of the menstrual cycle, we assessed T‐cell activation in the female genital tract (FGT) using CMCS and in the blood through PBMCS. We also characterized regulatory T cells in the blood.

#### 3.3.1 Mucosal Compartment

At the female genital tract, the expression of CCR5 on CD8+ T cells was higher in HESN versus NN during the luteal phase of the menstrual cycle (*p* = 0.004; padj = 0.070, Figure 1b). There was no difference between menstrual cycle phases within each group.

#### 3.3.2 Blood

In the blood during the luteal phase, the per‐cell expression of CCR5 and HLA‐DR on CD8+ T cells was higher in HESN women compared to NN (*p* = 0.006; padj = 0.070, Figure 1c). However, HESN women showed a lower per‐cell HLA‐DR expression (*p* = 0.005; padj = 0.070) on CD8+ T cells at the luteal phase when compared to NN (Figure 1c).  In NN, the expression of the inhibitory marker CTLA‐4 was lower during the luteal phase (*p* = 0.001; padj = 0.028), whereas there was no change in CTLA‐4 expression in HESN. The Treg function marker CD39 expression was higher in HESN vs. NN during the luteal phase of the menstrual cycle (*p* = 0.003; padj = 0.058, Figure 1d).

### 3.4 NK Cells Profile

NK cells were characterized in the blood. Their phenotyping was conducted in two ways: 1) classic gating of CD16++CD56+ (mainly cytotoxic) and CD16+CD56++ (mainly cytokine‐producing) NK cells, and 2) using clustering algorithms to determine co‐expression of multiple phenotyping markers across transitional NK cell subsets. The classic gating strategy revealed that the proportion of NKG2D was lower during the luteal phase compared to the follicular phase in HESN women for CD16++CD56+ NK cells (*p* = 0.004; padj = 0.055, Figure 1e) and CD16+CD56++ NK cells (*p *= 0.001; padj = 0.020, Figure 1f). Additionally, the proportion of CD16+CD56++ NK cells expressing NKG2D, as well as its expression per cell, was reduced in New Neg women during the follicular phase (proportion *p* = 0.001; padj = 0.008, expression *p* = 0.008; padj = 0.054, Figure 1f). No changes were observed during the luteal phase of the menstrual cycle.

Finally, employing clustering algorithms (Supplemental Figure S2), eight distinct NK cell clusters were identified, with one cluster (cluster 6) significantly more abundant in HESN compared to NN during the follicular phase (*p* = 0.002; padj = 0.015, Supplemental Figure S2e). This cluster was identified as CD95+HLA‐DR+ NK cells. These findings, along with the NKG2D results, suggest that HESN women exhibit increased NK cell activation compared to NN during the follicular phase of the menstrual cycle.

## 4 Discussion

The immune quiescence (IQ) profile observed in HESN is characterized by a lower level of mucosal inflammation [14, 30] and baseline T cell activation [15, 31, 32]. However, how their immune system is affected by the menstrual cycle remains mostly unknown. To provide some answers to this important question, we have designed a study to look at the variations in NK and T cell populations of FSWs, and to compare them between HESN and FSWs newly involved in sex work (NN). Interestingly, we observed that HESN FSWs have a unique immune profile in response to the menstrual cycle.

Herein, out of all the soluble parameters analyzed, only the mucosal expression of Annexin A3 differed, with it being elevated during the luteal phase in HESN. Annexin A3 is an immune protein that regulates inflammation and cell migration [33, 34]. Our data suggests that HESN may have an advantage in controlling cell migration during the luteal phase of the menstrual cycle, potentially decreasing the number of HIV target cells and therefore, susceptibility to HIV infection.

It has been shown that mucosal inflammation significantly increases the risk of acquiring HIV as it brings activated T cells into the genital tract [35]. Activated CD4+ T cells are more susceptible to HIV infection compared to quiescent T cells and, once infected, produce up to 1000 times more virus [36]. In HESN, we observed increased blood expression of the functional marker CD39 on Treg during the luteal phase. Tregs expressing CD39+ suppress T cell proliferation and the production of inflammatory cytokines more effectively than Tregs that don't express CD39 [37]. While we did not see differences in the CD4+ T cell expression of HLA‐DR, HESN had lower expression of HLA‐DR on their CD8+ at luteal compared to NN, suggesting a better control of immune activation in HESN. In NN, we observed that Treg had lower expression of CTLA‐4 during the luteal phase of the menstrual cycle. CTLA‐4 expression is essential for Tregs to control the immune system [38]. This may suggest that while in HESN, there is an increase in the control of the immune system during the luteal phase of the menstrual cycle, NN have a lower ability to inhibit inflammation.

However, it is worth noting that, compared to a study by Oertelt‐Prigione, our study did not observe a decrease in the proportion of Treg between the phases of the menstrual cycle [39]. This could be because our study was conducted among FSWs, and sex work is an important immunomodulator [10]. Therefore, how immune cells respond to endogenous sex hormones in the context of sex work can be different in FSWs compared to women from the general population. Our data show that HESN responds differently to the natural fluctuation of sex hormones observed during the menstrual cycle, compared to NN, by having a greater ability to control cell activation during the luteal phase. The luteal phase, specifically 7–10 days post‐ovulation, has been associated with a window of susceptibility for HIV infection [40]. During that window, Weinberg et al. found a decrease in cell‐mediated immunity, which allows fertilization and the implantation of the ovum [41]. The different cellular activation responses to the natural variation in sex hormones between HESN and NN could be another mechanism to explain the natural resistance to HIV infection observed among some HESNs.

The HESN IQ phenotype has been associated with lower baseline CD4 T cell activation and vaginal inflammation, as well as higher levels of NK cell activation. In a cohort from Montreal, Canada, HESNs who used intravenous drugs were more likely to possess two copies of the KIR3DS1 compared to HIV‐infected drug users [42]. The expression of the activation marker NKG2D on NK cells has also been associated with the HESN phenotype in two cohorts of serodiscordant couples from India and FSWs from Benin [43, 44]. Our studies observed that HESN had a higher proportion of NK cells carrying NKG2D than NN (on CD16+CD56++ and CD16++CD56+ cells). However, for the first time, we observed that the proportion of NK cells expressing NKG2D changed according to the menstrual cycle phases in HESN but not in NN individuals. In HESN, both the cytokine‐producing and cytotoxic NK populations expressing NKG2D were elevated during the follicular phase of the menstrual cycle compared to the luteal phase. Our clustering algorithms also showed that HESN had higher levels of CD95+HLA‐DR+NK during the follicular phase, which suggests that in HESN, the innate response is more activated during the follicular phase of the menstrual cycle compared to during the luteal phase. Interestingly, among NN, there were no changes in these markers during the menstrual cycle. Our study provides novel insights into how the immune response of FSWs is affected by the menstrual cycle. Compared to the NN, HESN have a more activated NK profile during the follicular phase and a better control of T cell activation at the luteal phase of the menstrual cycle.

One limitation of our study is that it represents only one complete menstrual cycle and self‐reported date since last menses was used to help determine the phase of the cycle. Also, because of the low number of cells from cytobrushes, we could not look at the NK and Treg populations in the endocervix. Furthermore, it is possible that some of the participants in the NN groups would eventually progress into the HESN group and, therefore, could bias some effects. It is also possible that the effects measured here are due to the duration of sex work and not the HESN phenotype, though, due to our definition of HESN, this is impossible to untangle. Finally, due to the high number of markers and comparisons done, we used the Benjamini–Hochberg correction. Because our study is a discovery one, and because we had a small sample size, and there are limited data on the menstrual cycle's impact on FSWS, we decided to present all the results with an adjusted *p* value of < 0.075.

To our knowledge, our study is among the first to suggest that the immune system of HESN FSWs reacts differently to sex hormones. This unique response allows for better control of their mucosal immune activation during the luteal phase of the menstrual cycle, which has been identified as the window of susceptibility.

## Authors Contributions

Conceptualization: Julie Lajoie, Julius Oyugi, Joshua Kimani, Keith R. Fowke, and Kristina Broliden. Data curation: Monika M. Kowatsch, Kenneth Omollo, Frideborg Bradley, Anna Månberg, Peter Nilsson, Sofia Bergström, Julie Lajoie. Formal analysis: Monika M. Kowatsch, Frideborg Bradley, and Julie Lajoie. Funding acquisition: Keith R. Fowke, Kristina Broliden, and Julie Lajoie. Methodology: Monika M. Kowatsch, Kenneth Omollo, Frideborg Bradley, Anna Månberg, Peter Nilsson, Sofia Bergström, and Julie Lajoie. Project administration: Julie Lajoie, Keith R. Fowke, and Kristina Broliden. Supervision: Frideborg Bradley, Julius Oyugi, Keith R. Fowke, Julie Lajoie, and Kristina Broliden. Writing—original draft: Monika M. Kowatsch and Julie Lajoie. Writing—review and editing: Monika M. Kowatsch, Kenneth Omollo, Frideborg Bradley, Anna Månberg, Peter Nilsson, Sofia Bergström, Julius Oyugi, Joshua Kimani, Kristina Broliden, Keith R. Fowke, and Julie Lajoie. All authors have read and agreed to the published version of the manuscript.

## Policy on Using ChatGPT and Similar AI Tools

During the preparation of this work, M. K. and J. L. used Grammarly to edit the grammar and English syntax of the text. After using these tools, the author(s) reviewed and edited the content as needed and take full responsibility for the content of the manuscript.

## Funding

Funding support was provided by the Canadian Institutes of Health Research CIHR (MOP #86721 KRF) (MOP # 60172 JL) and the Swedish Research Council (VR‐V 2021‐02671, KB). The project also received funding from the European Union's Horizon 2020 Research and Innovation program under grant agreement no. 847943 (MISTRAL; KB, ADB).

## Ethics Statement

The study was approved by The Ethical Review Board of the Kenyatta National Hospital and University of Nairobi; The Ethical Review Board of the University of Manitoba; The Regional Ethical Review Board in Stockholm. The studies were conducted in accordance with the local legislation and institutional requirements. The participants provided their written informed consent to participate in this study.

## Conflicts of Interest

The authors have no conflicts of interest to declare.

## Supporting information




**Supplemental Table 1**: Flow cytometry markers used for immune cell phenotyping.
**Supplemental Table 2**: Table of un‐adjusted p values and adjusted p values.
**Supplemental Figure 1**: Gating strategy used for identification and characterization of a) T cells activation panel in PBMCs. b) Treg cells in PBMCs. c) T cell activation in CMCs. And d) NK cells in PBMCs.
**Supplemental Figure 2**: Comparison of NK cell clusters in HESN and New Negs between follicular and luteal phases of the menstrual cycle. a) heatmap of cluster identities, b) line graph of cluster identities, c) proportion of cells per cluster, d) overlay of clusters on reduction algorithms used, e) comparisons of clusters between HESN and New Neg women in follicular and luteal phases. All analysis was performed on log of parameter due to non‐normal distribution and mixed effects models were used to control for age and cycle length, solid symbols represent normal cycle length (between 26 and 32 days) and open symbols represent abnormal cycle length (not between 26 and 32 days). Samples from follicular phase are in blue circles and samples from luteal phase are in red triangles. Un‐adjusted *p* values <0.05 were considered significant, false discovery rate was applied by sample type and values <0.075 were retained and presented here with unadjusted *p* values. Full list of *p* values and sample type can be found in supplemental table 1. Cluster 6 was identified as HLA‐DR+CD95+ NK cells.

**Supporting File 1:** aji70198‐sup‐0002‐FigureS1.png

**Supporting FIle 2:** aji70198‐sup‐0003‐FigureS2.png

**Supporting FIle 3:** aji70198‐sup‐0004‐TableS1.csv

**Supporting FIle 4:** aji70198‐sup‐0005‐TableS2.docx

Supporting FIle 5: aji70198‐sup‐0006‐TableS1.pdf

## Data Availability

The authors have nothing to report.
