# Supplementary material for: HIV‐Exposed Seronegative Female Sex Workers Show Different Cellular Immune Profiles Across the Menstrual Cycle
Source: Am J Reprod Immunol. 2025 Dec 19;94(6):e70198. doi: 10.1111/aji.70198 (PMC12716113; doi:10.1111/aji.70198)
Supplement: Supplementary file 1 — Supplemental Table 1: Flow cytometry markers used for immune cell phenotyping. Supplemental Table 2: Table of un‐adjusted p values and adjusted p values. Supplemental Figure 1: Gating strategy used for identification and characterization of a) T cells activation panel in PBMCs. b) Treg cells in PBMCs. c) T cell activation in CMCs. And d) NK cells in PBMCs. Supplemental Figure 2: Comparison of NK cell clusters in HESN and New Negs between follicular and luteal phases of the menstrual cycle. a) heatmap of cluster identities, b) line graph of cluster identities, c) proportion of cells per cluster, d) overlay of clusters on reduction algorithms used, e) comparisons of clusters between HESN and New Neg women in follicular and luteal phases. All analysis was performed on log of parameter due to non‐normal distribution and mixed effects models were used to control for age and cycle length, solid symbols represent normal cycle length (between 26 and 32 days) and open symbols represent abnormal cycle length (not between 26 and 32 days). Samples from follicular phase are in blue circles and samples from luteal phase are in red triangles. Un‐adjusted p values <0.05 were considered significant, false discovery rate was applied by sample type and values <0.075 were retained and presented here with unadjusted p values. Full list of p values and sample type can be found in supplemental table 1. Cluster 6 was identified as HLA‐DR+CD95+ NK cells. [file AJI-94-e70198-s002.docx]

**Supplemental figure and table caption**

**Supplemental Table 1.** Flow cytometry markers used for immune cell phenotyping.

**Supplemental Table 2.** Table of un-adjusted p values and adjusted p values.

**Supplemental Figure 1. Gating strategy used for identification and characterization of a) T cells actiation panel in PBMCs. b) Treg cells in PBMCs. c) T cell activation in CMCs. And d) NK cells in PBMCs.**

**Supplemental Figure 2.** Comparison of NK cell clusters in HESN and New Negs between follicular and luteal phases of the menstrual cycle. a) heatmap of cluster identities, b) line graph of cluster identities, c) proportion of cells per cluster, d) overlay of clusters on reduction algorithms used, e) comparisons of clusters between HESN and New Neg women in follicular and luteal phases. All analysis was performed on log of parameter due to non-normal distribution and mixed effects models were used to control for age and cycle length, solid symbols represent normal cycle length (between 26 and 32 days) and open symbols represent abnormal cycle length (not between 26 and 32 days). Samples from follicular phase are in blue circles and samples from luteal phase are in red triangles. Un-adjusted p values <0.05 were considered significant, false discovery rate was applied by sample type and values <0.075 were retained and presented here with unadjusted p values.. Full list of p values and sample type can be found in supplemental table 1. Cluster 6 was identified as HLA-DR+CD95+ NK cells.
