# Supplementary figures and images for: HIV‐Exposed Seronegative Female Sex Workers Show Different Cellular Immune Profiles Across the Menstrual Cycle
Source: Am J Reprod Immunol. 2025 Dec 19;94(6):e70198. doi: 10.1111/aji.70198 (PMC12716113; doi:10.1111/aji.70198)

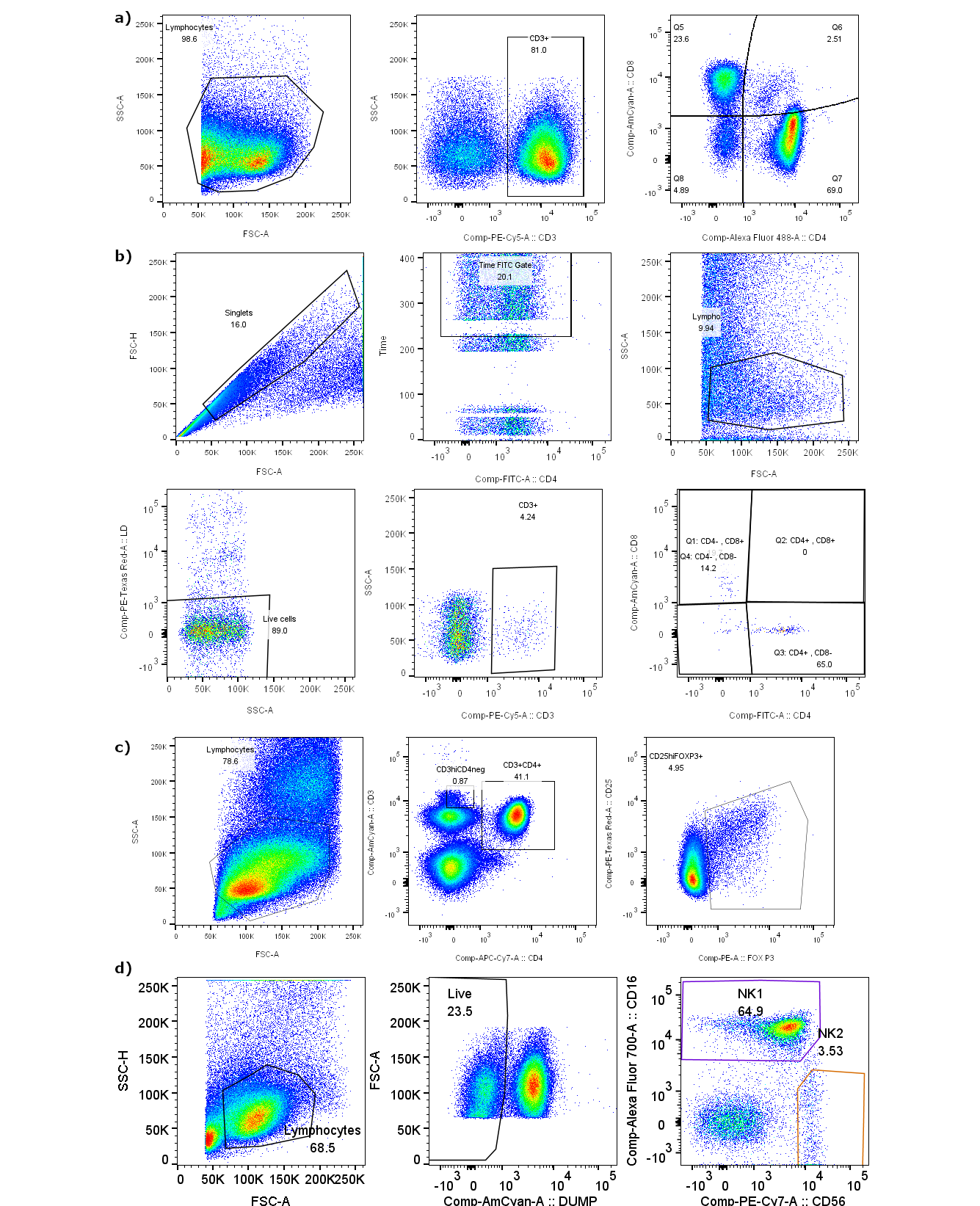

Supplement: Supplementary file 2 — Supporting File 1: aji70198‐sup‐0002‐FigureS1.png [file AJI-94-e70198-s005.png]

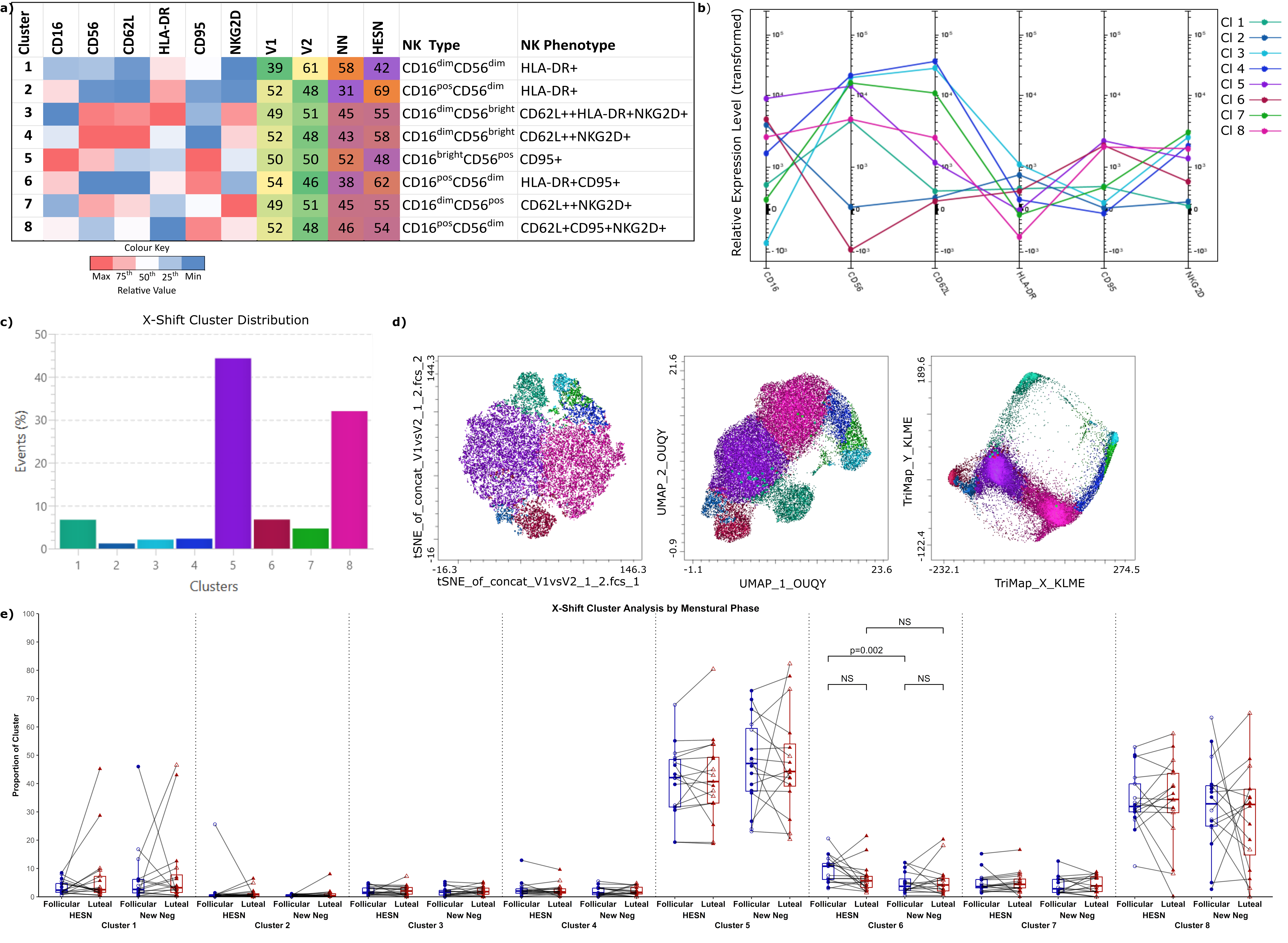

Supplement: Supplementary file 3 — Supporting FIle 2: aji70198‐sup‐0003‐FigureS2.png [file AJI-94-e70198-s001.png]
