# Supplementary material for: HIV‐Exposed Seronegative Female Sex Workers Show Different Cellular Immune Profiles Across the Menstrual Cycle
Source: Am J Reprod Immunol. 2025 Dec 19;94(6):e70198. doi: 10.1111/aji.70198 (PMC12716113; doi:10.1111/aji.70198)
Supplement: Supplementary file 5 — Supporting FIle 4: aji70198‐sup‐0005‐TableS2.docx [file AJI-94-e70198-s006.docx]

| **Marker** | **Fluorophore** | **Clone** | **Manufacterer** |
| --- | --- | --- | --- |
| **T cell characterization on PBMCs and CMCs** | | | |
| CD3 | PE-Cy5 | UCHT1 | BD Biosciences, USA |
| CD8 | V500 |  | BD Biosciences, USA |
| CD4 | FITC | RPA-T4 | BD Biosciences, USA |
| CD161 | APC | DX12 | BD Biosciences, USA |
| CCR5 | V450 | 2D7/CCR5 | BD Biosciences, USA |
| CD69 | PE-Cy7 | FN50 | BD Biosciences, USA |
| CD95 | PE | DX2 | BD Biosciences, USA |
| HLA-DR | APC-H7 | L243 | BD Biosciences, USA |
| Live Dead discriminant | Far Red | NAN | Invitrogen, USA |
| **T reg cell phenotyping on PBMCs** | | | |
| CD3 | PE.Cy5 | UCHT1 | BD Biosciences, USA |
| CD4 | APC-Cy7 | SK3 | Biolegend |
| CD25 | PE-CF594 | M0A251 | BD Biosciences, USA |
| CD127 | PeCy7 | HIL-7R-M21 | BD Biosciences, USA |
| FoxP3 | PE | PCH101 | eBioscience |
| Helios | APC | 22F6 | eBioscience |
| CTLA-4 | PECy5 | BNI3 | BD Biosciences, USA |
| CD39 | BV421 | A1 | Biolegend |
| Integrin- β7 | FITC | F1B504 | BD Biosciences, USA |
| Live Dead discriminant | Far Red | NAN | Invitrogen, USA |
| **NK cell phenotyping on PBMCs** |  |  |  |
| CD45 | Pe-Cy5 | HI30 | BD Biosciences, USA |
| CD3 | V500 | UCHT1 | BD Biosciences, USA |
| CD14 | V500 | M5E2 | BD Biosciences, USA |
| CD19 | V500 | HIB19 | BD Biosciences, USA |
| CD16 | Alexa-700 | 3G8 | BD Biosciences, USA |
| CD56 | Pe-Cy7 | B159 | BD Biosciences, USA |
| CD62L | APC | DREG-56 | Biolegend |
| CD57 | FITC | NK-1 | BD Biosciences, USA |
| CD95 | PE | DX2 | BD Biosciences, USA |
| HLA-DR | APC-H7 | L243 | BD Biosciences, USA |
| NKG2D | PE-CF594 | NKL | BD Biosciences, USA |
| CCR5 | V450 | 2D7/CCR5 | BD Biosciences, USA |
| Live Dead discriminant | Far Red | NAN | Invitrogen, USA |
