# Supplementary material for: HIV‐Exposed Seronegative Female Sex Workers Show Different Cellular Immune Profiles Across the Menstrual Cycle
Source: Am J Reprod Immunol. 2025 Dec 19;94(6):e70198. doi: 10.1111/aji.70198 (PMC12716113; doi:10.1111/aji.70198)
Supplement: Supplementary file 6 — Supporting FIle 5: aji70198‐sup‐0006‐TableS1.pdf [file AJI-94-e70198-s004.pdf]

FDR_Group	Variable	Follicular_HESN	Follicular_New Neg	Luteal_HESN	Luteal_New Neg	p.value_HESN	p_adj_HESN	p.value_New Neg	p_adj_New Neg	p.value_Follicular	p_adj_Follicular	p.value_Luteal	p_adj_Luteal
Cytokines	Plasma_IFNy	5.37 [0.535 to 12.97]	5.89 [0.4 to 16.25]	4.44 [0.4 to 14.16]	7.125 [3.9 to 16.32]	0.865	0.938	0.305	0.715	0.738	0.833	0.793	0.94
Cytokines	Plasma_IL10	0.55 [0.55 to 0.55]	0.55 [0.55 to 0.55]	0.55 [0.55 to 0.55]	0.55 [0.55 to 0.55]	0.081	0.472	0.783	0.884	0.391	0.77	0.814	0.94
Cytokines	Plasma_IL12_p70	4.3 [0.3 to 9.075]	0.3 [0.3 to 4.75]	4.66 [0.3 to 9.73]	3.175 [0.3 to 9.615]	0.887	0.938	0.33	0.718	0.286	0.77	0.801	0.94
Cytokines	Plasma_sCD40L	2296.97 [1651.87 to 3609.37]	1581.59 [1250.86 to 2623.15]	1622.06 [1354.787 to 3108.835]	1482.225 [1015.832 to 2332.62]	0.044	0.409	0.256	0.715	0.973	0.973	0.912	0.94
Cytokines	Plasma_IL17A	3.69 [0.35 to 8.793]	3.86 [0.35 to 12.415]	1.23 [0.35 to 6.293]	3.44 [0.35 to 11.23]	0.461	0.735	0.994	0.998	0.744	0.833	0.462	0.855
Cytokines	Plasma_IL1a	4.7 [4.7 to 18.21]	4.7 [4.7 to 4.78]	4.7 [4.7 to 4.7]	4.7 [4.7 to 5.308]	0.262	0.597	0.562	0.777	0.414	0.77	0.682	0.94
Cytokines	Plasma_IL1b	0.4 [0.4 to 3.85]	0.4 [0.4 to 0.4]	0.4 [0.4 to 1.64]	0.4 [0.4 to 1.855]	0.571	0.735	0.435	0.748	0.239	0.77	0.867	0.94
Cytokines	Plasma_IL2	0.5 [0.5 to 0.5]	0.5 [0.5 to 0.5]	0.5 [0.5 to 0.5]	0.5 [0.5 to 0.5]	0.058	0.433	0.689	0.822	0.765	0.833	0.312	0.792
Cytokines	Plasma_IL8	10.41 [6.29 to 25.825]	8.32 [6.54 to 13.18]	7.99 [6.26 to 29.06]	8.65 [5.938 to 14.142]	0.359	0.698	0.455	0.748	0.385	0.77	0.448	0.855
Cytokines	Plasma_IP10	604.12 [465.735 to 888.195]	475.53 [409.91 to 738.115]	657.69 [430.7 to 886.03]	492.68 [348.715 to 726.355]	0.576	0.735	0.707	0.831	0.432	0.77	0.2	0.617
Cytokines	Plasma_MCP1	257.47 [210.35 to 320.02]	249.07 [201.435 to 291.205]	247.35 [189.14 to 307.55]	255.975 [229.982 to 303.172]	0.274	0.597	0.495	0.748	0.179	0.77	0.545	0.924
Cytokines	Plasma_MIP1a	1.45 [1.45 to 11.008]	1.45 [1.45 to 5.352]	1.45 [1.45 to 17.66]	1.45 [1.45 to 6.83]	0.444	0.735	0.762	0.881	0.645	0.822	0.364	0.792
Cytokines	Plasma_MIP1b	44.26 [30.245 to 63.81]	32.98 [27.025 to 38.775]	40.6 [29.37 to 59.51]	31.84 [19.302 to 39.542]	0.029	0.409	0.788	0.884	0.048	0.587	0.116	0.581
Cytokines	Plasma_TNFa	13.3 [6.395 to 15.7]	9.26 [7.295 to 11.005]	8.82 [5.38 to 15.78]	10.535 [6.42 to 12.3]	0.243	0.597	0.166	0.659	0.482	0.77	0.599	0.924

Cytokines	Plasma_IL1RA	19.37 [5.085 to 61.28]	17.34 [5.19 to 30.505]	29.84 [4.15 to 72.78]	22.305 [4.768 to 31.59]	0.496	0.735	0.616	0.814	0.569	0.78	0.349	0.792
Cytokines	Plasma_MIG	920.11 [576.585 to 1133.783]	853 [472.73 to 1148.15]	798.88 [594.29 to 977.62]	601.88 [366.033 to 896.24]	0.349	0.698	0.176	0.659	0.52	0.77	0.034	0.581
Cytokines	Plasma_MIP3a	15.16 [9.29 to 18.47]	5.15 [0.8 to 16.59]	14.4 [10.14 to 20.7]	12.23 [1.888 to 25.683]	0.495	0.735	0.466	0.748	0.099	0.612	0.125	0.581
Cytokines	Plasma_IL2RA	878 [615.5 to 1356.5]	2200 [893 to 2887]	861 [587.25 to 1266]	2234 [1362 to 2792]	0.981	0.981	0.53	0.769	0.044	0.587	0.103	0.581
Cytokines	Cv_IFNy	0.4 [0.4 to 0.615]	0.4 [0.4 to 5]	0.4 [0.4 to 7.05]	0.4 [0.4 to 0.4]	0.115	0.473	0.068	0.638	0.363	0.77	0.151	0.581
Cytokines	Cv_IL10	0.55 [0.55 to 0.55]	0.55 [0.55 to 0.55]	0.55 [0.55 to 2.04]	0.55 [0.55 to 0.55]	0.153	0.548	0.444	0.748	0.93	0.956	0.064	0.581
Cytokines	Cv_IL12_p70	3.94 [0.3 to 7.045]	4.29 [0.3 to 12.12]	1.82 [0.3 to 7.787]	0.3 [0.3 to 3.345]	0.764	0.891	0.016	0.638	0.812	0.859	0.114	0.581
Cytokines	Cv_IL15	0.6 [0.6 to 0.6]	0.6 [0.6 to 2.085]	0.6 [0.6 to 0.6]	0.6 [0.6 to 0.6]	0.55	0.735	0.088	0.638	0.678	0.822	0.14	0.581
Cytokines	Cv_sCD40L	2.55 [2.55 to 3.405]	2.55 [2.55 to 4.16]	2.55 [2.55 to 4.415]	2.55 [2.55 to 2.55]	0.508	0.735	0.129	0.638	0.405	0.77	0.588	0.924
Cytokines	Cv_IL17A	0.35 [0.35 to 3.612]	0.35 [0.35 to 4.755]	0.35 [0.35 to 4.805]	0.35 [0.35 to 0.35]	0.497	0.735	0.08	0.638	0.486	0.77	0.173	0.581
Cytokines	Cv_IL1a	34.52 [9.78 to 217.075]	96.92 [25.46 to 381.97]	103.555 [48.198 to 200.438]	150.985 [73.65 to 551.33]	0.089	0.472	0.295	0.715	0.597	0.789	0.886	0.94
Cytokines	Cv_IL1b	0.4 [0.4 to 25.475]	7.7 [0.4 to 94.96]	12.76 [0.552 to 29.197]	9 [1.183 to 23.477]	0.163	0.548	0.904	0.98	0.25	0.77	0.919	0.94
Cytokines	Cv_IL2	0.5 [0.5 to 0.5]	0.5 [0.5 to 3.92]	0.5 [0.5 to 3.31]	0.5 [0.5 to 0.5]	0.567	0.735	0.324	0.718	0.505	0.77	0.453	0.855
Cytokines	Cv_IL8	231.1 [57.155 to 546.145]	1170.95 [376.73 to 2237.05]	700.715 [186.745 to 2759.1]	1528.295 [529.21 to 2371.042]	0.042	0.409	0.953	0.98	0.012	0.44	0.288	0.792
Cytokines	Cv_IP10	27.47 [6.44 to 128.915]	48.76 [21.075 to 117.1]	50.89 [4.3 to 182.28]	94.695 [23.95 to 319.752]	0.835	0.936	0.238	0.715	0.52	0.77	0.161	0.581

Cytokines	Cv_MCP1	79.17 [14.445 to 146.53]	29.36 [14.69 to 179.6]	26.62 [4.028 to 66.363]	17.175 [4.932 to 69.048]	0.104	0.473	0.04	0.638	0.434	0.77	0.759	0.94
Cytokines	Cv_MIP1a	1.45 [1.45 to 11.15]	9.11 [1.45 to 20.595]	5.945 [1.45 to 23.215]	5.765 [1.45 to 12.087]	0.216	0.572	0.489	0.748	0.092	0.612	0.94	0.94
Cytokines	Cv_MIP1b	9.27 [2.405 to 17.475]	14.73 [8.1 to 24.18]	8.185 [1.5 to 19.952]	7.48 [2.973 to 18.087]	0.975	0.981	0.281	0.715	0.226	0.77	0.802	0.94
Cytokines	Cv_TNFa	0.35 [0.35 to 0.35]	0.35 [0.35 to 2.045]	0.35 [0.35 to 0.35]	0.35 [0.35 to 0.35]	0.717	0.884	0.666	0.822	0.568	0.78	0.647	0.94
Cytokines	Cv_IL1RA	32808.965 [3900 to 75473.238]	73152.15 [39544.175 to 159168.08]	77056.5 [32203.85 to 167221.395]	83590.935 [39109.99 to 216601.088]	0.016	0.409	0.803	0.887	0.174	0.77	0.599	0.924
Cytokines	Cv_MIG	94.38 [5.15 to 336.58]	195.3 [61.805 to 503.9]	173.495 [5.15 to 768.89]	308.365 [53.727 to 2239.398]	0.215	0.572	0.998	0.998	0.077	0.612	0.357	0.792
Cytokines	Cv_MIP3a	0.8 [0.8 to 8.135]	0.8 [0.8 to 0.8]	0.8 [0.8 to 42.295]	0.8 [0.8 to 11.575]	0.179	0.553	0.491	0.748	0.689	0.822	0.907	0.94
Cytokines	Cv_IL2RA	3 [3 to 26.25]	3 [3 to 3]	3 [3 to 16]	3 [3 to 3]	0.771	0.891	0.276	0.715	0.371	0.77	0.166	0.581
GenT	Rat_CMC_CD4CD8	1.522 [1.35 to 2.462]	1.708 [0.849 to 2.766]	2.495 [1.849 to 3.834]	1.763 [1.119 to 1.846]	0.224	0.838	0.965	0.965	0.807	0.807	0.366	0.732
GenT	DP	0.9 [0.44 to 1.51]	0.82 [0.54 to 1.27]	0.84 [0.42 to 1.392]	0.98 [0.6 to 1.07]	0.838	0.838	0.843	0.963	0.484	0.646	0.321	0.732
GenT	DN	5.19 [4.06 to 8.35]	6.01 [3.76 to 7.98]	5.055 [3.845 to 8.285]	5.2 [4.075 to 10.315]	0.82	0.838	0.182	0.794	0.334	0.646	0.871	0.871
GenT	Rat_PBMC_CD4CD8	3.059 [2.09 to 3.667]	2.425 [2.006 to 3.245]	2.732 [2.115 to 5.729]	2.87 [2.252 to 3.521]	0.662	0.838	0.414	0.794	0.246	0.646	0.776	0.871
CD8	Cv_CD8	32.4 [20 to 35.8]	33 [19.25 to 38.4]	26.7 [17.75 to 29.6]	32.95 [28.075 to 40]	0.318	0.985	0.716	0.923	0.555	0.891	0.555	0.859

CD8	Cv_CD8CCR5	54.7 [45.4 to 62.2]	52.7 [24.2 to 68.1]	64.85 [52.55 to 77.65]	27.45 [19.05 to 31.65]	0.812	0.985	0.311	0.884	0.616	0.904	0.413	0.859
CD8	Cv_CD8CD69	39.7 [18.65 to 55.85]	59.4 [34.3 to 71.8]	26.2 [14.65 to 51.65]	40.7 [26.35 to 44.775]	0.719	0.985	0.28	0.884	0.986	0.986	0.566	0.859
CD8	Cv_CD8CD95	94.1 [85.3 to 96]	92.1 [83.2 to 96.3]	95.8 [93.375 to 98.525]	96.35 [51.225 to 97.75]	0.612	0.985	0.441	0.884	0.879	0.962	0.238	0.859
CD8	Cv_CD8CD161	13.3 [9.942 to 26.275]	21.4 [16.8 to 30.2]	19.15 [10.465 to 25.475]	13.5 [12.475 to 15.575]	0.901	0.985	0.71	0.923	0.512	0.891	0.462	0.859
CD8	Cv_CD8CD161pp	5.06 [1.685 to 8.1]	5.13 [0.45 to 8.815]	5.035 [3.128 to 11.05]	4.405 [2.09 to 8.46]	0.551	0.985	0.834	0.936	0.562	0.891	0.332	0.859
CD8	Cv_CD8HLADR	13.6 [10 to 18.1]	15.4 [9.165 to 19.8]	13.4 [8.235 to 17.925]	15.45 [13.175 to 16.975]	0.874	0.985	0.681	0.923	0.928	0.962	0.733	0.86
CD8	Cv_CD8CCR5pCD69n	20.9 [13.4 to 27.525]	12.5 [3.57 to 26.3]	28.8 [11.835 to 37.25]	12.93 [5.61 to 21.975]	0.626	0.985	0.319	0.884	0.638	0.904	0.353	0.859
CD8	Cv_CD8CCR5pCD69p	13.95 [2.562 to 32.725]	4.72 [0 to 33.3]	13 [3.04 to 20.85]	22.4 [10.495 to 33.675]	0.968	0.985	0.2	0.884	0.317	0.891	0.626	0.859
CD8	Cv_CD8CCR5nCD69p	14.15 [7.252 to 19.825]	12.5 [4.05 to 32.2]	10.3 [5.745 to 12.15]	19.75 [15.275 to 23.475]	0.396	0.985	0.468	0.884	0.516	0.891	0.422	0.859
CD8	Cv_CD8CCR5nCD69n	42.1 [24.8 to 63.975]	58.1 [18.8 to 65.8]	43.3 [21.35 to 63.25]	51.75 [28.975 to 61.25]	0.585	0.985	0.434	0.884	0.716	0.937	0.919	0.919
CD8	CD8	23.6 [20.4 to 29.9]	28 [21.2 to 31.6]	24.3 [14.15 to 30.625]	23.9 [19 to 28.65]	0.72	0.985	0.316	0.884	0.233	0.891	0.817	0.877
CD8	CD8CCR5	17 [11.5 to 24]	11.8 [7.45 to 20.4]	16.75 [8.433 to 22.05]	16.5 [8.415 to 23.175]	0.946	0.985	0.36	0.884	0.75	0.944	0.686	0.859
CD8	CD8CD69	19.2 [12.8 to 29]	14.4 [8.2 to 27.9]	19.5 [10.035 to 35.35]	14.9 [6.545 to 26.2]	0.985	0.985	0.496	0.893	0.892	0.962	0.495	0.859

CD8	CD8CD95	68.7 [58.4 to 81.4]	70.1 [65.4 to 77.7]	72.95 [64.175 to 85.425]	69.8 [61.7 to 75.6]	0.317	0.985	0.19	0.884	0.787	0.955	0.376	0.859
CD8	CD8CD161	8.28 [5.3 to 13.5]	6.3 [5.66 to 12.8]	11.95 [7.345 to 21.625]	8.85 [7.15 to 14.1]	0.25	0.985	0.268	0.884	0.455	0.891	0.453	0.859
CD8	CD8CD161 pp	3.25 [1.67 to 9.44]	2.91 [1.98 to 5.17]	5.12 [1.485 to 8.337]	3.99 [2.08 to 9.85]	0.708	0.985	0.407	0.884	0.933	0.962	0.851	0.877
CD8	CD8HLADR	5.66 [3.57 to 9.49]	3.36 [2.09 to 5.56]	5.435 [3.53 to 7.97]	4.24 [1.975 to 5.26]	0.62	0.985	0.98	0.98	0.056	0.554	0.109	0.741
CD8	CD8TCM	11.7 [8.86 to 14.7]	7.86 [4.71 to 12.2]	14.1 [10.325 to 18.025]	8.46 [6.845 to 12.3]	0.728	0.985	0.31	0.884	0.067	0.554	0.21	0.859
CD8	CD8naive	35.6 [28.2 to 50.1]	29.2 [20.7 to 46.3]	35.9 [23.75 to 45.375]	36.6 [31.5 to 43.1]	0.436	0.985	0.668	0.923	0.256	0.891	0.669	0.859
CD8	CD8Teff	19.5 [14.4 to 32.9]	22.3 [10.5 to 35.4]	20.8 [13.925 to 32.5]	22.6 [14.9 to 30.5]	0.533	0.985	0.843	0.936	0.909	0.962	0.848	0.877
CD8	CD8TEM	21.5 [14.4 to 26.9]	24.3 [19.3 to 42.1]	23.5 [15.4 to 32.025]	25 [17.85 to 31.7]	0.543	0.985	0.942	0.971	0.305	0.891	0.604	0.859
CD8	Cv_CD8MFI CCR5	580 [499 to 715]	611 [500 to 702]	590 [579 to 694]	461 [455 to 521]	0.039	0.444	0.016	0.265	0.381	0.891	0.004	0.07
CD8	Cv_CD8MFI CD69	1994 [1417 to 3275]	2144.5 [1640.75 to 2505]	1509 [1300.25 to 2125.75]	1332.5 [1209 to 1507]	0.137	0.93	0.015	0.265	0.704	0.937	0.145	0.822
CD8	Cv_CD8MFI CD95	1709 [1335 to 2010]	1564 [1185 to 2169]	2131.5 [1792 to 2408.75]	2006 [1658.5 to 2050.5]	0.028	0.444	0.707	0.923	0.441	0.891	0.023	0.195
CD8	Cv_CD8MFI CD161	1924.5 [1804.75 to 2310.25]	1839 [1613.5 to 2114]	2117.5 [1813 to 2352.75]	1971 [1827.75 to 2089.5]	0.873	0.985	0.342	0.884	0.2	0.891	0.817	0.877
CD8	Cv_CD8MFI CD161pp	6975 [4986 to 10451]	6322 [4038.25 to 7760]	6934 [5849 to 7826.75]	5604 [5439 to 6432]	0.928	0.985	0.675	0.923	0.434	0.891	0.363	0.859

CD8	Cv_CD8MFIHLADR	975 [872.5 to 1176.25]	843.5 [703.75 to 1021.5]	1002 [816 to 1220]	912 [797.5 to 934.25]	0.794	0.985	0.912	0.969	0.264	0.891	0.392	0.859
CD8	CD8MFICCR5	431 [385 to 480]	358 [304 to 390]	395 [355 to 462.75]	316 [304.75 to 374.25]	0.695	0.985	0.57	0.923	0.007	0.122	0.006	0.07
CD8	CD8MFICD69	2033 [1791 to 2529]	2206 [1822 to 2438]	1935 [1709.25 to 2273.25]	2056 [1757.5 to 2327.5]	0.354	0.985	0.239	0.884	0.504	0.891	0.646	0.859
CD8	CD8MFICD95	1888 [1495 to 2185]	1881 [1564 to 2419]	2035.5 [1516 to 2348.25]	1738 [1354.5 to 1949.5]	0.743	0.985	0.064	0.867	0.577	0.891	0.708	0.859
CD8	CD8MFICD161	2235 [2090 to 2389]	2397 [2020 to 2508]	2365.5 [2220.25 to 2715.75]	2222 [2154.5 to 2624]	0.123	0.93	0.45	0.884	0.23	0.891	0.603	0.859
CD8	CD8MFI_CD161pp	10942 [8901 to 14292]	9454 [8249 to 10462]	10701 [9379 to 11827.75]	8986 [8348.5 to 9766.5]	0.016	0.444	0.286	0.884	0.003	0.095	0.275	0.859
CD8	CD8MFIHLADR	1285 [1211 to 1394]	1358 [1204 to 2177]	1211.5 [1103.75 to 1341.25]	1529 [1405.5 to 2182.5]	0.483	0.985	0.394	0.884	0.081	0.554	0.005	0.07
CD4	Cv_CD4	53.7 [47.4 to 64]	56.1 [39.35 to 63.85]	64.35 [56.3 to 71.65]	52.15 [41.375 to 60.075]	0.264	0.884	0.764	0.905	0.323	0.781	0.253	0.472
CD4	Cv_CD4CCR5	48.9 [36.2 to 60.7]	42.7 [21.5 to 52.3]	44.25 [32.075 to 66.25]	33.35 [16.95 to 41.875]	0.742	0.884	0.501	0.774	0.903	0.939	0.362	0.539
CD4	Cv_CD4CD69	37.8 [16.8 to 41.2]	46.3 [17.35 to 58.5]	22.1 [10.5 to 34.05]	21.9 [15.075 to 30.225]	0.623	0.884	0.065	0.509	0.783	0.887	0.556	0.652
CD4	Cv_CD4CD95	88.9 [78.1 to 96.4]	90 [74.25 to 98.6]	95.3 [90.525 to 99.2]	97.6 [79.375 to 99.325]	0.053	0.884	0.821	0.905	0.499	0.788	0.488	0.607
CD4	Cv_CD4CD161	41.8 [33.7 to 56.175]	50 [31.05 to 58.8]	44.3 [29.625 to 57.275]	33.35 [20.925 to 51.7]	0.945	0.945	0.243	0.59	0.654	0.884	0.215	0.429

CD4	Cv_CD4HLADR	7.21 [5.56 to 16.1]	9.76 [5.505 to 27.2]	5.79 [4.478 to 11.775]	7.215 [3.675 to 8.805]	0.305	0.884	0.014	0.509	0.749	0.884	0.212	0.429
CD4	Cv_CD4CCR5pCD69n	12.8 [8.495 to 29.375]	11.4 [2.44 to 18.7]	15 [7.29 to 20.85]	14.15 [4.98 to 24.775]	0.274	0.884	0.485	0.774	0.508	0.788	0.5	0.607
CD4	Cv_CD4CCR5pCD69p	14.45 [2.395 to 27.725]	4.88 [0 to 25.8]	6.14 [2.87 to 13.9]	14.145 [8.838 to 19.625]	0.78	0.884	0.061	0.509	0.26	0.737	0.411	0.559
CD4	Cv_CD4CCR5nCD69p	17.15 [8.685 to 22.55]	14.8 [2.49 to 22.2]	11.8 [5.81 to 17.9]	7.4 [3.768 to 20.505]	0.371	0.884	0.965	0.97	0.038	0.431	0.114	0.389
CD4	Cv_CD4CCR5nCD69n	48.05 [28.725 to 63.875]	66.7 [31.8 to 76.2]	58.1 [41.7 to 76.25]	58.6 [38.2 to 64.15]	0.494	0.884	0.728	0.905	0.676	0.884	0.601	0.659
CD4	CD4	70.3 [62.4 to 74.5]	65.8 [63 to 68.8]	65.95 [61.85 to 74.2]	68.5 [62.15 to 69.85]	0.514	0.884	0.902	0.948	0.345	0.781	0.66	0.701
CD4	CD4CCR5	5.665 [3.607 to 11.625]	3.89 [2.735 to 8.515]	5.36 [3.322 to 10.26]	6.525 [4.102 to 9.938]	0.724	0.884	0.395	0.725	0.939	0.939	0.727	0.749
CD4	CD4CD69	7.64 [2.35 to 14.3]	7.39 [2.46 to 12.7]	6.835 [4.562 to 13.675]	5.14 [2.1 to 10.54]	0.711	0.884	0.669	0.887	0.712	0.884	0.277	0.472
CD4	CD4CD95	69.1 [62 to 74.3]	66.5 [56.9 to 72.6]	67.9 [64.15 to 76.05]	66.1 [56.7 to 69.3]	0.559	0.884	0.059	0.509	0.855	0.937	0.477	0.607
CD4	CD4CD161	18.1 [13.1 to 27.6]	15.1 [10.6 to 20.9]	22.9 [18.925 to 27.075]	12.7 [9.105 to 16.1]	0.044	0.884	0.825	0.905	0.53	0.788	0.017	0.191
CD4	CD4HLADR	2.83 [2.12 to 4.06]	2.25 [1.42 to 3.05]	2.925 [1.928 to 3.763]	1.87 [1.445 to 2.835]	0.618	0.884	0.657	0.887	0.114	0.522	0.13	0.397
CD4	CD4TCM	52.1 [43.7 to 56.8]	43.5 [41.4 to 50.6]	52.7 [47.3 to 59.975]	47.6 [42.4 to 56.7]	0.354	0.884	0.55	0.823	0.462	0.788	0.345	0.539
CD4	CD4naive	28.1 [18.8 to 32.1]	26.4 [8.99 to 33.6]	27.1 [19.375 to 34.275]	25.1 [21.1 to 34.05]	0.652	0.884	0.426	0.762	0.155	0.528	0.595	0.659

CD4	CD4Teff	0.62 [0.34 to 0.97]	0.95 [0.37 to 1.84]	0.425 [0.332 to 1.06]	1.15 [0.655 to 2.22]	0.672	0.884	0.473	0.774	0.135	0.522	0.101	0.381
CD4	CD4TEM	21 [13.4 to 23]	23.5 [18.7 to 32.4]	17.45 [14.475 to 22.375]	24.6 [17.35 to 27.1]	0.384	0.884	0.18	0.509	0.016	0.291	0.045	0.191
CD4	Tconv_B7	28.4 [25.7 to 40.4]	25.6 [23 to 30.2]	32.7 [25.95 to 39.1]	26.15 [18.325 to 33.25]	0.93	0.945	0.839	0.905	0.017	0.291	0.025	0.191
CD4	Tconv_CD25	14.1 [11.05 to 22.75]	11.6 [8.975 to 14.8]	15.85 [11.45 to 21.575]	9.81 [7.185 to 11.1]	0.765	0.884	0.109	0.509	0.088	0.522	0.006	0.191
CD4	Cv_CD4MFICCR5	579.5 [506 to 720.25]	636 [543 to 674]	582.5 [550 to 687.75]	428.5 [414.5 to 552.75]	0.375	0.884	0.095	0.509	0.919	0.939	0.029	0.191
CD4	Cv_CD4MFICD69	2214 [1946 to 2725.5]	2428.5 [2025.5 to 3874]	1811 [1678.5 to 3111]	2007.5 [1951.75 to 2045.25]	0.832	0.912	0.111	0.509	0.491	0.788	0.273	0.472
CD4	Cv_CD4MFICD95	3427 [2476 to 4074]	3305 [2420 to 4872.5]	3420.5 [3180.25 to 3989.75]	3003.5 [2409.5 to 3449.75]	0.293	0.884	0.47	0.774	0.754	0.884	0.16	0.401
CD4	Cv_CD4MFICD161	2914 [2602.5 to 3163.75]	3352 [2613.5 to 4041]	2903 [2592 to 3492.25]	3061.5 [2349.25 to 3688.25]	0.097	0.884	0.13	0.509	0.317	0.781	0.395	0.559
CD4	Cv_CD4MFIHLADR	1532 [1125 to 1761]	1212 [910.25 to 1549.5]	1175.5 [969 to 1493.5]	1232.5 [1039 to 4503.25]	0.485	0.884	0.073	0.509	0.383	0.788	0.165	0.401
CD4	CD4MFICCR5	354.5 [312.75 to 406.75]	304 [289 to 346]	347.5 [320.25 to 408]	295.5 [282 to 332]	0.573	0.884	0.777	0.905	0.098	0.522	0.035	0.191
CD4	CD4MFICD69	2025 [1630 to 2292]	2231 [2080 to 2782]	1691 [1440 to 2287.75]	2193 [1920.5 to 2491]	0.327	0.884	0.155	0.509	0.063	0.522	0.14	0.397
CD4	CD4MFICD95	3182 [2843 to 3511]	3046 [2563 to 3918]	3376 [2798.75 to 3863]	2804 [2312 to 3126]	0.902	0.945	0.029	0.509	0.563	0.798	0.365	0.539

CD4	CD4MFICD161	3043 [2835 to 3590]	3187 [3064 to 3462]	3133 [2848.5 to 3291.25]	3474 [3066 to 3616]	0.642	0.884	0.146	0.509	0.533	0.788	0.037	0.191
CD4	CD4MFIHLADR	1448 [1342 to 1567]	1570 [1273 to 2266]	1364.5 [1121.25 to 1576]	1521 [1433 to 2110]	0.398	0.884	0.326	0.711	0.138	0.522	0.184	0.418
CD4	Tconv_MFIB7	1179 [1139.5 to 1392]	1168 [1111.5 to 1270.5]	1378 [1163 to 1581.5]	1147.5 [1041.75 to 1180.25]	0.689	0.884	0.373	0.717	0.172	0.53	0.042	0.191
CD4	Tconv_MFICD25	1193 [1153 to 1229]	1197 [1153 to 1251]	1202.5 [1151.5 to 1232.5]	1181 [1137.5 to 1228.5]	0.394	0.884	0.286	0.67	0.417	0.788	0.918	0.918
Treg	Tr_Freq	4.8 [3.73 to 5.625]	4.02 [3.545 to 4.93]	5.14 [4.26 to 5.735]	3.88 [3.3 to 5.27]	0.558	0.788	0.405	0.636	0.847	0.916	0.198	0.612
Treg	Tr_B7	15.7 [12.7 to 18.85]	14.3 [10.9 to 17.75]	19.7 [14.1 to 22.3]	13.1 [8.56 to 18]	0.158	0.402	0.529	0.729	0.174	0.696	0.009	0.081
Treg	Tr_CD39hi	47.3 [35.35 to 64.55]	48.2 [39.85 to 54.05]	44.35 [39.825 to 57.4]	42.4 [23.285 to 48.2]	0.038	0.222	0.053	0.249	0.634	0.869	0.488	0.725
Treg	Tr_CD39lo	31.8 [25.35 to 44.15]	36 [27.95 to 42.65]	32.2 [26.7 to 43.825]	43.5 [30.85 to 50.35]	0.291	0.582	0.074	0.253	0.841	0.916	0.332	0.664
Treg	Tr_CTLA4	63 [53.65 to 67.25]	63 [54.8 to 68.1]	57.3 [53.2 to 64.725]	55.5 [44.15 to 59.4]	0.071	0.314	0.065	0.253	0.38	0.869	0.271	0.637
Treg	Tr_Hel	64.9 [55.15 to 73.3]	65.1 [60.2 to 73.45]	58.9 [51.075 to 67]	67.3 [49.35 to 76.95]	0.039	0.222	0.482	0.676	0.493	0.869	0.214	0.612
Treg	Tr_Freq_Hel	4.19 [3.355 to 5.385]	4.13 [3.415 to 4.7]	4.3 [2.83 to 4.7]	3.68 [2.768 to 5.46]	0.109	0.364	0.392	0.627	0.797	0.911	0.981	0.981
Treg	Tr_Hel_B7	18.9 [15.6 to 22.5]	16.7 [12.85 to 21.85]	24.15 [18.05 to 28.35]	14.85 [10.688 to 20]	0.234	0.492	0.415	0.639	0.08	0.401	0.005	0.064
Treg	Tr_Hel_CD39hi	46.2 [34.25 to 64.1]	50.1 [36.3 to 58.95]	46.6 [40 to 57.8]	45.7 [10.072 to 48.625]	0.091	0.332	0.076	0.253	0.915	0.934	0.709	0.873

Treg	Tr_Hel_CD39hiCTLA4	23.9 [16.5 to 33.8]	27.1 [21 to 37.55]	22.5 [17.5 to 28.6]	20.6 [4.75 to 23.8]	0.012	0.12	0	0.019	0.443	0.869	0.863	0.923
Treg	Tr_Hel_CD39lo	34.6 [25.4 to 46.25]	35.1 [27.15 to 44.1]	36.8 [30 to 43.8]	44.5 [30.875 to 47.35]	0.177	0.402	0.225	0.457	0.921	0.934	0.83	0.923
Treg	Tr_Hel_CTLA4	48.2 [44.25 to 56.45]	51.7 [46.75 to 58.65]	48.6 [42.9 to 52]	43.2 [38.35 to 47.5]	0.181	0.402	0.147	0.406	0.576	0.869	0.422	0.725
Treg	Tr_Fp3Helneg	2.55 [1.67 to 3.605]	2.24 [1.79 to 2.915]	3 [2.24 to 4.29]	1.87 [1.732 to 3.287]	0.812	0.877	0.758	0.858	0.483	0.869	0.314	0.664
Treg	Tr_Fp3Heln_B7	19.4 [17 to 24.2]	18.1 [13.6 to 22.85]	22.65 [17.3 to 23.5]	18.1 [16.4 to 20.975]	0.798	0.877	0.562	0.763	0.51	0.869	0.775	0.886
Treg	Tr_Fp3Heln_CD39hi	35.7 [14.5 to 40.55]	25.8 [17.5 to 36.05]	32 [20.9 to 49.1]	19.75 [3.72 to 29.625]	0.474	0.782	0.258	0.457	0.363	0.869	0.23	0.613
Treg	Tr_Fp3Heln_CD39lo	34.7 [29.8 to 50.4]	35.4 [24.9 to 49.25]	36.7 [26.5 to 42.3]	42.8 [35.4 to 55.775]	0.882	0.916	0.186	0.438	0.656	0.869	0.143	0.612
Treg	Tr_Fp3Heln_CTLA4	65 [51.2 to 70.45]	59.8 [49.3 to 63.75]	59.5 [51.2 to 67.6]	51.25 [45.775 to 59.475]	0.174	0.402	0.144	0.406	0.522	0.869	0.378	0.693
Treg	Tr_Fp3Heln_Hel	0.99 [0.465 to 2.67]	1.72 [0.54 to 4.71]	1.2 [1.01 to 2.89]	1.88 [0.868 to 3.315]	0.163	0.402	0.152	0.407	0.078	0.401	0.742	0.873
Treg	activeTreg	1.55 [1.265 to 2.71]	2.05 [1.515 to 2.625]	1.92 [1.38 to 2.35]	1.42 [1.168 to 1.85]	0.783	0.877	0.044	0.243	0.934	0.934	0.127	0.612
Treg	restingTreg	1.13 [0.99 to 1.435]	1.15 [0.79 to 1.33]	1.07 [0.68 to 1.66]	1.585 [1.272 to 1.863]	0.607	0.788	0.257	0.457	0.654	0.869	0.256	0.637
Treg	nonTreg	3.99 [2.89 to 5.415]	3.73 [3.075 to 4.64]	4.04 [3.58 to 5.02]	3.215 [2.938 to 4.327]	0.503	0.782	0.344	0.573	0.461	0.869	0.328	0.664
Treg	Tr_HelFp3neg	0.93 [0.675 to 1.425]	0.83 [0.58 to 1.055]	0.91 [0.55 to 1.47]	1.08 [0.9 to 1.345]	0.662	0.803	0.244	0.457	0.324	0.869	0.726	0.873

Treg	Tr_MFIB7	1095 [990 to 1423.5]	1366 [1209 to 1519.5]	1059 [974 to 1261]	1199 [954 to 1237]	0.066	0.314	0.046	0.243	0.145	0.644	0.381	0.693
Treg	Tr_MFI_CD39hi	5103 [4070.5 to 6114.5]	4537 [4159 to 4932]	5189 [3923.25 to 6543.5]	4903 [3989.5 to 5659.5]	0.893	0.916	0.884	0.929	0.59	0.869	0.652	0.873
Treg	Tr_MFI_CD39lo	952 [865 to 1159.5]	853 [713.5 to 1051.5]	918 [854.5 to 1070.25]	657 [608 to 782.5]	0.445	0.773	0.046	0.243	0.029	0.397	0.003	0.058
Treg	Tr_MFI_CTLA4	2341 [2223 to 2810.5]	2629 [2277 to 2848.5]	2229 [2079.25 to 2396]	2219 [1929.5 to 2428.5]	0.006	0.12	0.001	0.028	0.429	0.869	0.665	0.873
Treg	Tr_MFI_Fp3	2087 [1726.5 to 2387]	2083 [1659.5 to 2642.5]	2095 [1778.5 to 2453.25]	1487 [1348 to 1966.5]	0.641	0.801	0.021	0.153	0.717	0.869	0.171	0.612
Treg	Tr_MFI_Hel	2810 [2513 to 3077]	2766 [2556 to 3079.5]	2332 [2150.75 to 2929.5]	2583 [1948 to 2843]	0.011	0.12	0.029	0.194	0.793	0.911	0.877	0.923
Treg	Tr_Hel_MFI_B7	937 [867.5 to 1432]	1312 [1164 to 1515]	1077 [1024.5 to 1182]	1215.5 [1091.5 to 1273]	0.508	0.782	0.135	0.4	0.021	0.397	0.202	0.612
Treg	Tr_Hel_MFI_CD39hi	4682 [4078.5 to 6154.5]	4594 [4120.5 to 4974]	5147 [4066 to 6516]	4832 [3743.25 to 5802.5]	0.389	0.707	0.905	0.929	0.705	0.869	0.489	0.725
Treg	Tr_Hel_MFI_CD39lo	1000 [851 to 1172]	903 [766.5 to 1025]	978 [895 to 1051]	665.5 [609.5 to 807.25]	0.578	0.788	0.014	0.12	0.044	0.397	0.001	0.033
Treg	Tr_Hel_MFI_CTLA4	2021 [1769 to 2249]	2341 [2016.5 to 2553.5]	1957 [1811 to 2111]	2071.5 [1795 to 2159.5]	0.032	0.222	0.003	0.074	0.06	0.4	0.553	0.791
Treg	Tr_Hel_Fp3MFI	1881 [1600 to 2251]	2111 [1628.5 to 2455]	1860 [1652 to 2279]	1396.5 [1276.75 to 1768.5]	0.765	0.877	0.009	0.105	0.449	0.869	0.16	0.612
Treg	Tr_Hel_MFI_Fp3	2837 [2675.5 to 3049]	2788 [2481 to 3077.5]	2328 [2160 to 2894]	2446 [1906.75 to 2817.75]	0.012	0.12	0.018	0.145	0.695	0.869	0.927	0.951

Treg	Tr_Fp3Heln_MFIB7	1567 [1346.5 to 1753.5]	1646 [1529.5 to 1952]	1636.5 [1344.25 to 1932.25]	1419.5 [1179.75 to 1579]	0.972	0.972	0.012	0.115	0.387	0.869	0.129	0.612
Treg	Tr_Fp3Heln_MFICD39hi	4343 [3926 to 5311]	4041 [3732.5 to 4537]	4751 [3886 to 5688]	4099.5 [3825.25 to 4737.25]	0.376	0.707	0.823	0.886	0.666	0.869	0.485	0.725
Treg	Tr_Fp3Heln_MFICD39lo	871 [740.5 to 1077]	765 [566 to 931.5]	860 [753 to 943]	624 [557 to 745.25]	0.611	0.788	0.192	0.439	0.033	0.397	0.01	0.081
Treg	Tr_Fp3Heln_MFICTLA4	2599 [2107 to 3008.5]	2728 [2414 to 3028]	2405 [2060 to 2599]	2360 [1845 to 2779.75]	0.085	0.332	0.005	0.081	0.353	0.869	0.742	0.873
Treg	Tr_Fp3Heln_MFI_Fp3	1509 [1359.5 to 1655]	1413 [1242 to 1677]	1561 [1258 to 1731]	1308.5 [1188.5 to 1410.75]	0.178	0.402	0.008	0.105	0.359	0.869	0.069	0.459
Treg	Tr_Fp3Heln_MFIHel	1025 [1014.5 to 1042]	1024 [973 to 1036]	1038 [1026 to 1049]	1024 [1019 to 1042.5]	0.567	0.788	0.069	0.253	0.05	0.397	0.47	0.725
NK	P_Live.x	25.9 [16.65 to 35.6]	16.3 [10.9 to 21.2]	23.1 [16.975 to 29.075]	19.1 [14.1 to 25.35]	0.97	0.97	0.526	0.666	0.337	0.337	0.629	0.629
NK1	P_NK1	22.2 [14.4 to 40.85]	38.1 [17.1 to 48.8]	24.35 [12.775 to 33.2]	30.6 [14.15 to 45]	0.609	0.656	0.369	0.811	0.201	0.312	0.365	0.806
NK1	P_CCR5NK1	1.29 [0.462 to 3.728]	1.54 [0.355 to 4.17]	1.03 [0.242 to 5.305]	1.015 [0.43 to 2.598]	0.462	0.656	0.582	0.858	0.152	0.267	0.711	0.905
NK1	P_CD57NK1	61.3 [54.6 to 68.95]	60.7 [48.5 to 65.1]	60.35 [51.225 to 73.65]	63.3 [56.125 to 65.575]	0.578	0.656	0.956	0.964	0.481	0.519	0.611	0.887
NK1	P_CD62LNK1	33.2 [27.9 to 36.25]	34.7 [16.1 to 45.1]	38.2 [31.425 to 46.075]	31.15 [23.675 to 43.225]	0.568	0.656	0.403	0.811	0.123	0.246	0.816	0.909
NK1	P_CD95NK1	59.4 [43.15 to 69.5]	50.7 [41.9 to 59.5]	54.2 [36.1 to 70.25]	43.5 [31.75 to 53.925]	0.286	0.573	0.066	0.618	0.45	0.519	0.214	0.75

NK1	P_HLADRNK1	9.72 [5.82 to 13.55]	4.75 [3.53 to 7.65]	7.06 [5.043 to 12.25]	4.42 [3.438 to 7.04]	0.397	0.656	0.292	0.811	0.087	0.205	0.073	0.341
NK1	P_NKG2DNK1	43.5 [36.8 to 53.45]	32.4 [24 to 45.2]	34.75 [24.5 to 40.575]	40.5 [22.225 to 45.525]	0.004	0.055	0.826	0.913	0.038	0.176	0.597	0.887
NK2	P_NK2	1.67 [1.175 to 3.995]	3.22 [2.03 to 4.4]	2.54 [1.455 to 3.428]	2.75 [1.665 to 3.88]	0.366	0.64	0.197	0.959	0.067	0.235	0.72	0.82
NK2	P_CCR5NK2	7.12 [3.942 to 11.35]	5.37 [2.49 to 8.41]	4.66 [1.685 to 14.475]	4.56 [2.43 to 7.345]	0.153	0.64	0.802	0.959	0.257	0.4	0.746	0.82
NK2	P_CD57NK2	1.09 [0.37 to 1.665]	0.72 [0.29 to 1.91]	1.09 [0.485 to 3.042]	0.76 [0.235 to 2.38]	0.269	0.64	0.878	0.959	0.656	0.706	0.151	0.526
NK2	P_CD62LNK2	93.9 [89.45 to 96.3]	86.1 [63.6 to 95.1]	93.9 [82.6 to 97.125]	92.1 [52.45 to 97]	0.231	0.64	0.461	0.959	0.117	0.279	0.761	0.82
NK2	P_CD95NK2	8.24 [5.715 to 19.55]	7.2 [4.76 to 12.4]	9.485 [3.963 to 13.55]	5.51 [3.145 to 6.345]	0.487	0.758	0.561	0.959	0.133	0.279	0.188	0.526
NK2	P_HLADRNK2	5.12 [2.42 to 9.235]	4.79 [2.92 to 10.8]	3.975 [2.555 to 7.44]	3.57 [2.41 to 5.805]	0.708	0.88	0.052	0.73	0.436	0.509	0.491	0.764
NK2	P_NKG2DNK2	82.6 [73.95 to 87.3]	65.4 [52 to 81.8]	69.3 [59.75 to 80.4]	64.1 [52.75 to 77.45]	0.001	0.02	0.849	0.959	0.001	0.008	0.154	0.526
NK1	MFI_NK1	14891 [10423.5 to 17678.5]	12240 [9197 to 14383]	14152 [9974 to 16118]	10895 [8328 to 14949]	0.276	0.573	0.964	0.964	0.088	0.205	0.387	0.806
NK1	MFI_CCR5NK1	646 [641 to 672]	686 [646.75 to 862.25]	663 [644 to 685]	656.5 [643 to 766.5]	0.14	0.573	0.133	0.728	0.008	0.108	0.844	0.909
NK1	MFI_CD57NK1	20764 [16007 to 24889.5]	22502 [14483 to 31263]	17554.5 [14095.75 to 23337]	23989 [17450 to 30782.75]	0.271	0.573	0.458	0.811	0.456	0.519	0.069	0.341
NK1	MFI_CD62LNK1	7095 [5964 to 10271.5]	7397 [6559 to 8834]	7808.5 [5623.5 to 8751.75]	7019.5 [5796.5 to 8112.5]	0.511	0.656	0.782	0.913	0.226	0.317	0.633	0.887

NK1	MFI_CD95 NK1	1964 [1774.5 to 2189]	1811 [1652 to 1931]	1854.5 [1677.25 to 2185.75]	1636 [1570 to 1769.5]	0.273	0.573	0.12	0.728	0.036	0.176	0.019	0.266
NK1	MFI_HLADR NK1	3305 [2780 to 4256.5]	3145 [2670 to 3678]	3558.5 [2647.5 to 4248.25]	2995.5 [2324.25 to 3515.5]	0.82	0.82	0.454	0.811	0.812	0.812	0.403	0.806
NK1	MFI_NKG2DNK1	1621 [1552.5 to 1779]	1478 [1405 to 1615]	1495 [1440.5 to 1618.75]	1509.5 [1358 to 1587.25]	0.015	0.106	0.666	0.913	0.05	0.177	0.949	0.949
NK2	MFI_NK2	18030 [14858 to 20424.5]	13828 [12381 to 16192]	16671.5 [13538.5 to 18976.25]	12611 [11977 to 16849]	0.325	0.64	0.538	0.959	0.026	0.121	0.064	0.526
NK2	MFI_CCR5 NK2	550.5 [526 to 593.25]	587 [511.5 to 629]	562 [516 to 646]	559 [511 to 641]	0.88	0.88	0.794	0.959	0.75	0.75	0.943	0.943
NK2	MFI_CD57 NK2	2998 [1061.5 to 22544]	3705 [1017 to 9266.75]	4913 [1188 to 19622]	2457 [1136.5 to 14508]	0.823	0.88	0.537	0.959	0.225	0.393	0.387	0.677
NK2	MFI_CD62L NK2	28072 [23912 to 33446]	27673 [15819 to 33678]	29517 [25277 to 31793]	25458 [12679 to 32099]	0.811	0.88	0.995	0.995	0.378	0.481	0.555	0.777
NK2	MFI_CD95 NK2	923 [837 to 1024]	916 [836 to 1009]	897.5 [838.25 to 1038.75]	875 [810 to 950.25]	0.549	0.769	0.449	0.959	0.315	0.44	0.272	0.543
NK2	MFI_HLADR NK2	2734 [2206 to 6989]	2578 [2181 to 2894]	2452 [2042.5 to 5933.5]	2245.5 [2032.25 to 2604.25]	0.348	0.64	0.656	0.959	0.139	0.279	0.257	0.543
NK2	MFI_NKG2DNK2	1917 [1669.5 to 2198]	1512 [1290 to 1946]	1686 [1475.75 to 2078]	1478 [1367 to 1601.5]	0.019	0.135	0.28	0.959	0.008	0.054	0.107	0.526
MDR	Mense_XS_p1	2.35 [1.57 to 4.71]	2.59 [1.372 to 6.16]	2.51 [1.57 to 7.22]	3.215 [1.53 to 7.732]	0.505	0.683	0.739	0.957	0.676	0.773	0.887	0.887
MDR	Mense_XS_p2	0.47 [0.16 to 0.78]	0.39 [0.16 to 0.78]	0.78 [0.16 to 1.1]	0.39 [0.12 to 1.1]	0.212	0.683	0.893	0.957	0.89	0.89	0.177	0.305

MDR	Mense_XS_p3	1.57 [1.1 to 3.14]	1.645 [0.59 to 2.277]	2.04 [0.94 to 3.3]	1.805 [0.903 to 3.06]	0.337	0.683	0.473	0.957	0.305	0.61	0.411	0.47
MDR	Mense_XS_p4	2.04 [1.26 to 2.83]	1.41 [0.702 to 3.06]	1.73 [1.26 to 2.83]	1.57 [0.983 to 3.375]	0.598	0.683	0.348	0.957	0.245	0.61	0.118	0.305
MDR	Mense_XS_p5	42.1 [31.7 to 48.5]	47.1 [37.3 to 59.475]	40.7 [33.1 to 49.3]	44.25 [39.075 to 53.975]	0.8	0.8	0.865	0.957	0.248	0.61	0.267	0.356
MDR	Mense_XS_p6	10.8 [6.12 to 11.8]	3.69 [2.312 to 6.315]	5.65 [3.3 to 7.22]	4.16 [2.082 to 6.515]	0.044	0.349	0.933	0.957	0.002	0.015	0.123	0.305
MDR	Mense_XS_p7	3.61 [3.14 to 6.12]	2.825 [1.53 to 6.16]	4.4 [3.14 to 6.28]	3.845 [1.842 to 7.028]	0.493	0.683	0.232	0.957	0.414	0.663	0.191	0.305
MDR	Mense_XS_p8	31.9 [30 to 39.9]	32.85 [24.975 to 39.25]	34.4 [29.7 to 43.6]	32.6 [14.765 to 38.025]	0.589	0.683	0.197	0.957	0.56	0.747	0.188	0.305
Protein	Rn16_LRG1_HPA001888	5.269 [4.346 to 5.825]	5.67 [5.159 to 6.277]	5.215 [4.808 to 6.132]	5.303 [4.924 to 5.972]	0.655	0.714	0.499	0.941	0.186	0.396	0.783	0.939
Protein	Rn16_SPINK5_HPA009067	4.614 [4.263 to 5.456]	4.852 [4.599 to 5.692]	4.736 [4.331 to 5.811]	5.223 [4.62 to 5.564]	0.225	0.327	0.988	1	0.065	0.299	0.398	0.915
Protein	Rn16_KRT1_HPA019797	4.578 [4.334 to 5.376]	5.434 [4.849 to 7.129]	6.265 [4.678 to 7.134]	5.784 [4.721 to 7.97]	0.017	0.098	0.852	0.971	0.058	0.299	0.966	0.992
Protein	Rn16_CD5L_HPA026432	6.372 [6.307 to 6.408]	6.335 [6.245 to 6.364]	6.328 [6.195 to 6.365]	6.34 [6.282 to 6.392]	0.042	0.112	0.225	0.941	0.108	0.299	0.153	0.915
Protein	Rn16_CAMP_HPA029874	4.471 [4.293 to 4.675]	4.7 [4.494 to 5.074]	4.804 [4.417 to 5.228]	4.673 [4.428 to 5.204]	0.022	0.098	0.723	0.941	0.253	0.396	0.261	0.915
Protein	Rn16_PLG_HPA048823	4.435 [4.163 to 5.202]	4.828 [4.59 to 5.943]	4.7 [4.254 to 5.628]	4.876 [4.372 to 5.872]	0.5	0.598	0.902	0.982	0.069	0.299	0.277	0.915
Protein	Rn16_CFH_HPA049176	5.288 [4.241 to 5.903]	5.771 [4.872 to 6.169]	5.79 [4.721 to 6.137]	5.165 [4.819 to 6.255]	0.714	0.764	0.787	0.943	0.165	0.386	0.463	0.915

Protein	Rn16_LGMN_HPA000799	4.817 [4.357 to 5.379]	5.118 [4.855 to 5.823]	5.464 [4.775 to 6.1]	5.288 [4.624 to 5.924]	0.014	0.098	0.594	0.941	0.25	0.396	0.686	0.915
Protein	Rn16_MUC1_HPA004179	4.77 [4.248 to 5.371]	4.905 [4.505 to 5.413]	4.92 [4.59 to 5.368]	4.795 [4.613 to 5.24]	0.308	0.427	0.489	0.941	0.26	0.396	0.637	0.915
Protein	Rn16_MUC1_HPA007235	4.677 [4.562 to 4.998]	4.836 [4.654 to 5.167]	4.92 [4.609 to 5.214]	4.753 [4.627 to 5.202]	0.2	0.305	0.948	1	0.472	0.574	0.799	0.939
Protein	Rn16_S100A6_HPA007575	6.86 [4.444 to 7.374]	6.983 [6.039 to 7.9]	7.261 [6.338 to 7.931]	7.315 [6.636 to 7.542]	0.05	0.114	0.785	0.943	0.272	0.405	0.613	0.915
Protein	Rn16_CAPNS1_HPA006872	4.51 [3.866 to 5.159]	4.97 [4.35 to 5.657]	5.288 [4.492 to 6.23]	5.14 [4.795 to 6.068]	0.025	0.098	0.554	0.941	0.556	0.652	0.382	0.915
Protein	Rn16_ANXA1_HPA011271	6.539 [4.424 to 8.324]	7.575 [6.882 to 8.49]	7.519 [6.759 to 8.697]	8.271 [6.479 to 8.789]	0.052	0.114	0.625	0.941	0.097	0.299	0.735	0.915
Protein	Rn16_SPINK5_HPA011351	5.175 [4.472 to 6.152]	5.489 [4.867 to 6.971]	5.209 [4.736 to 6.64]	6.208 [5.27 to 6.83]	0.338	0.447	1	1	0.025	0.299	0.148	0.915
Protein	Rn16_ANXA3_HPA013398	5.18 [4.109 to 6.385]	6.36 [5.843 to 7.502]	7.162 [5.759 to 7.832]	6.524 [6.125 to 7.629]	0.001	0.061	0.765	0.943	0.04	0.299	0.412	0.915
Protein	Rn16_ANXA3_HPA013431	4.956 [4.144 to 5.826]	5.858 [5.529 to 7.056]	6.753 [5.274 to 7.378]	6.193 [5.786 to 7.252]	0.002	0.061	0.798	0.943	0.034	0.299	0.536	0.915
Protein	Rn16_TXNDC17_HPA022931	5.656 [4.979 to 6.434]	6.165 [5.76 to 6.712]	6.547 [5.96 to 6.901]	6.47 [6.295 to 6.868]	0.035	0.103	0.519	0.941	0.349	0.463	0.675	0.915
Protein	Rn16_S100A2_HPA034651	4.732 [4.432 to 4.952]	4.868 [4.663 to 5.116]	5.004 [4.5 to 5.46]	5.084 [4.827 to 5.691]	0.591	0.689	0.272	0.941	0.892	0.923	0.731	0.915
Protein	Rn16_LTF_HPA059976	4.709 [4.511 to 5.02]	5.187 [4.754 to 5.573]	5.136 [4.525 to 5.946]	5.236 [4.763 to 6.381]	0.037	0.103	0.488	0.941	0.085	0.299	0.643	0.915
Protein	Rn16_ITIH2_HPA062964	5.375 [4.786 to 6.302]	5.572 [5.11 to 6.084]	5.328 [4.895 to 6.479]	5.73 [5.087 to 6.389]	0.776	0.803	0.375	0.941	0.566	0.652	0.326	0.915

Protein	Rn16_BCAP31_HPA003906	4.653 [4.376 to 5.143]	4.956 [4.6 to 5.72]	4.905 [4.43 to 6.657]	5.225 [4.575 to 6.229]	0.009	0.098	0.348	0.941	0.288	0.409	0.685	0.915
Protein	Rn16_TTR_HPA005150	7.916 [7.329 to 8.452]	7.944 [7.592 to 8.541]	7.898 [7.268 to 8.197]	7.329 [7.071 to 8.124]	0.323	0.438	0.12	0.941	0.449	0.559	0.861	0.973
Protein	Rn16_NCF2_HPA002327	4.494 [4.428 to 4.713]	4.754 [4.527 to 5.084]	4.804 [4.394 to 5.515]	4.731 [4.516 to 5.274]	0.034	0.103	0.697	0.941	0.313	0.425	0.719	0.915
Protein	Rn16_AHSG_HPA001524	5.511 [3.997 to 6.558]	5.945 [5.23 to 6.953]	5.919 [4.283 to 6.405]	5.155 [4.44 to 6.539]	0.99	0.99	0.302	0.941	0.104	0.299	0.549	0.915
Protein	Rn16_MSN_HPA011135	4.769 [4.273 to 5.383]	5.215 [4.885 to 5.888]	5.147 [4.503 to 6.234]	5.296 [4.94 to 6.069]	0.046	0.114	0.716	0.941	0.07	0.299	0.724	0.915
Protein	Rn16_CSTB_HPA017380	5.551 [4.877 to 7.252]	6.654 [5.538 to 7.5]	6.17 [5.257 to 7.839]	6.717 [5.945 to 7.662]	0.351	0.447	0.579	0.941	0.118	0.313	0.239	0.915
Protein	Rn16_KLK10_HPA017195	5.16 [4.703 to 5.932]	5.733 [5.165 to 6.073]	5.455 [4.965 to 6.533]	5.683 [5.187 to 6.626]	0.122	0.22	0.504	0.941	0.221	0.396	0.669	0.915
Protein	Rn16_SERPINB1_HPA018871	4.923 [4.467 to 5.316]	5.231 [4.732 to 5.749]	5.407 [4.6 to 6.238]	5.293 [4.835 to 6.002]	0.104	0.193	0.515	0.941	0.48	0.574	0.926	0.992
Protein	Rn16_SERPINB5_HPA019132	4.58 [4.31 to 5.282]	5.333 [4.823 to 5.796]	5.609 [4.709 to 6.48]	5.717 [4.887 to 6.638]	0.079	0.155	0.24	0.941	0.179	0.396	0.443	0.915
Protein	Rn16_SERPINB5_HPA020136	4.736 [4.474 to 5.266]	5.056 [4.685 to 5.396]	5.407 [4.797 to 5.771]	5.122 [4.73 to 5.699]	0.599	0.689	0.47	0.941	0.825	0.881	0.703	0.915
Protein	Rn16_MMP8_HPA022935	5.063 [4.966 to 5.172]	5.17 [5.043 to 5.437]	5.361 [5.039 to 5.934]	5.204 [5.043 to 5.497]	0.012	0.098	0.399	0.941	0.683	0.758	0.349	0.915
Protein	Rn16_SPRR3_HPA024330	9.235 [8.954 to 9.658]	9.545 [9.353 to 9.64]	9.386 [9.173 to 9.64]	9.585 [9.484 to 9.646]	0.168	0.278	0.632	0.941	0.031	0.299	0.235	0.915
Protein	Rn16_IL18_HPA003980	5.007 [4.888 to 5.2]	5.081 [4.949 to 5.226]	5.198 [4.997 to 5.387]	5.144 [4.959 to 5.335]	0.619	0.699	0.502	0.941	0.375	0.477	0.536	0.915

Protein	Rn16_TTR_HPA002550	9.064 [8.925 to 9.204]	9.062 [8.877 to 9.129]	8.961 [8.849 to 9.115]	8.903 [8.856 to 9.041]	0.167	0.278	0.32	0.941	0.652	0.737	0.476	0.915
Protein	Rn16_S100P_HPA019502	4.902 [4.135 to 5.709]	5.481 [4.98 to 6.535]	5.984 [4.548 to 6.746]	5.882 [4.864 to 6.519]	0.021	0.098	0.959	1	0.074	0.299	0.815	0.939
Protein	Rn16_KRT1_HPA017917	4.716 [4.201 to 4.961]	4.727 [4.361 to 6.383]	5.142 [4.376 to 5.883]	5.432 [4.397 to 6.4]	0.189	0.296	0.579	0.941	0.053	0.299	0.205	0.915
Protein	Rn16_FLNA_HPA002925	4.335 [4.17 to 5.094]	4.984 [4.464 to 5.375]	4.585 [4.284 to 6.766]	5.288 [4.652 to 6.779]	0.047	0.114	0.18	0.941	0.209	0.396	0.512	0.915
Protein	Rn16_S100A12_HPA002881	7.638 [5.128 to 9.013]	8.76 [6.896 to 9.335]	8.814 [7.906 to 9.397]	8.529 [7.231 to 8.875]	0.028	0.099	0.837	0.964	0.209	0.396	0.519	0.915
Protein	Rn16_S100A12_HPA003620	6.778 [4.085 to 8.172]	7.857 [5.974 to 8.444]	7.765 [6.884 to 8.552]	7.669 [5.941 to 7.94]	0.035	0.103	0.792	0.943	0.285	0.409	0.502	0.915
Protein	Rn16_FGA_HPA051370	5.184 [4.575 to 7.21]	6.28 [5.34 to 7.244]	6.339 [4.59 to 6.719]	6.322 [5.44 to 6.918]	0.639	0.709	0.706	0.941	0.08	0.299	0.103	0.915
Protein	Rn16_FLNA_HPA001016	4.521 [4.324 to 5.084]	4.97 [4.625 to 5.254]	4.575 [4.394 to 6.402]	5.027 [4.713 to 6.349]	0.052	0.114	0.234	0.941	0.239	0.396	0.634	0.915
Protein	Rn16_SOD1_HPA001401	5.666 [4.921 to 6.13]	6.026 [5.485 to 6.424]	6.267 [5.781 to 6.577]	6.278 [6.016 to 6.492]	0.013	0.098	0.531	0.941	0.26	0.396	0.522	0.915
Protein	Rn16_FABP4_HPA002188	4.437 [4.234 to 4.653]	4.564 [4.382 to 4.994]	5.063 [4.433 to 5.582]	4.677 [4.369 to 5.366]	0.03	0.101	0.721	0.941	0.838	0.881	0.225	0.915
Protein	Rn16_S100A9_HPA004193	9.563 [9.222 to 9.763]	9.707 [9.569 to 9.813]	9.735 [9.651 to 9.782]	9.718 [9.598 to 9.829]	0.023	0.098	0.811	0.943	0.057	0.299	0.924	0.992
Protein	Rn16_CAPN1_HPA005992	4.436 [4.147 to 4.674]	4.663 [4.382 to 4.959]	4.796 [4.317 to 5.185]	4.754 [4.477 to 5.046]	0.096	0.183	0.556	0.941	0.199	0.396	0.453	0.915
Protein	Rn16_TMPRSS11D_HPA010927	4.584 [4.111 to 5.119]	4.844 [4.62 to 5.535]	5.165 [4.668 to 6.025]	4.955 [4.531 to 5.923]	0.014	0.098	0.683	0.941	0.202	0.396	0.601	0.915

Protein	Rn16_ECM1_HPA027241	6.165 [4.501 to 7.249]	7.271 [6.602 to 8.033]	7.499 [6.362 to 8.036]	7.151 [6.874 to 7.856]	0.023	0.098	0.593	0.941	0.022	0.299	0.598	0.915
Protein	Rn16_CCL5_HPA042290	4.537 [3.778 to 5.226]	4.277 [4.06 to 4.52]	4.277 [3.97 to 4.635]	4.086 [3.946 to 4.49]	0.298	0.423	0.916	0.989	0.985	0.985	0.417	0.915
Protein	Rn16_SPRR3_HPA044467	8.887 [8.581 to 9.368]	9.267 [8.969 to 9.34]	9.147 [8.767 to 9.414]	9.288 [9.138 to 9.391]	0.144	0.251	0.642	0.941	0.025	0.299	0.231	0.915
Protein	Rn16_CXCL9_HPA046604	4.06 [3.892 to 4.23]	4.127 [4.007 to 4.5]	4.19 [3.979 to 4.644]	4.207 [3.984 to 4.551]	0.063	0.133	0.412	0.941	0.149	0.379	0.61	0.915
Protein	Rn16_CORO1A_HPA051132	4.658 [4.174 to 6.065]	6.519 [4.779 to 7.092]	6.05 [5.504 to 7.415]	5.735 [5.155 to 6.763]	0.02	0.098	0.751	0.943	0.227	0.396	0.229	0.915
Protein	Rn16_CCL21_HPA051210	4.824 [4.6 to 5.041]	4.82 [4.677 to 5.161]	4.956 [4.666 to 5.287]	4.912 [4.764 to 5.175]	0.22	0.327	0.782	0.943	0.307	0.425	0.937	0.992
Protein	Rn16_TACSTD2_HPA055067	4.043 [3.911 to 5.007]	4.615 [4.158 to 4.795]	4.234 [3.96 to 5.659]	4.483 [4.073 to 5.178]	0.347	0.447	0.811	0.943	0.363	0.472	0.815	0.939
Protein	Rn16_HIST1H1B_HPA060331	4.404 [4.204 to 4.698]	4.625 [4.419 to 4.991]	4.625 [4.487 to 5.257]	5.007 [4.681 to 5.397]	0.076	0.155	0.209	0.941	0.103	0.299	0.308	0.915
Protein	Rn16_GPX3_HPA062579	4.856 [4.417 to 5.115]	4.828 [4.499 to 5.383]	5.03 [4.609 to 5.277]	4.692 [4.287 to 5.287]	0.878	0.893	0.972	1	0.947	0.963	0.992	0.992
Protein	Rn16_SLPI_HPA027774	5.501 [4.909 to 5.946]	5.838 [5.49 to 6.26]	6.221 [5.127 to 6.905]	6.012 [5.776 to 6.538]	0.008	0.098	0.293	0.941	0.157	0.384	0.988	0.992
Protein	Rn16_PI3_HPA017737	7.186 [5.797 to 8.169]	7.316 [5.779 to 8.698]	6.813 [5.723 to 8.297]	8.398 [6.687 to 8.662]	0.735	0.773	0.416	0.941	0.1	0.299	0.019	0.915
Protein	Rn16_TACSTD2_HPA043104	5.143 [4.841 to 5.457]	5.118 [4.874 to 5.493]	5.094 [4.846 to 5.328]	4.898 [4.812 to 5.312]	0.497	0.598	0.672	0.941	0.8	0.871	0.678	0.915
Protein	Rn16_CSTA_HPA000392	7.207 [6.617 to 7.883]	7.64 [7.417 to 8.133]	7.57 [7.097 to 8.02]	7.783 [7.282 to 8.095]	0.026	0.098	0.87	0.974	0.066	0.299	0.984	0.992

Protein	Rn16_CSTB _HPA058557	5.585 [4.872 to 7.62]	6.702 [5.04 to 7.501]	6.25 [5.143 to 8.013]	7.265 [5.523 to 8.045]	0.186	0.296	0.204	0.941	0.218	0.396	0.266	0.915
Protein	Rn16_HRG _HPA054598	4.95 [4.705 to 5.411]	5.545 [5.037 to 5.874]	5.1 [4.783 to 6.176]	5.381 [4.943 to 6.512]	0.489	0.598	0.506	0.941	0.043	0.299	0.065	0.915
